# Improving Ethanol Tolerance of *Escherichia coli* by Rewiring Its Global Regulator cAMP Receptor Protein (CRP)

**DOI:** 10.1371/journal.pone.0057628

**Published:** 2013-02-28

**Authors:** Huiqing Chong, Lei Huang, Jianwei Yeow, Ivy Wang, Hongfang Zhang, Hao Song, Rongrong Jiang

**Affiliations:** 1 School of Chemical & Biomedical Engineering, Nanyang Technological University, Singapore; 2 Institute of Biological Engineering, Department of Chemical and Biological Engineering, Zhejiang University, Hangzhou, P. R. China; 3 System Engineering, Life Technologies, Singapore; University of Nottingham, United Kingdom

## Abstract

A major challenge in bioethanol fermentation is the low tolerance of the microbial host towards the end product bioethanol. Here we report to improve the ethanol tolerance of *E. coli* from the transcriptional level by engineering its global transcription factor cAMP receptor protein (CRP), which is known to regulate over 400 genes in *E. coli*. Three ethanol tolerant CRP mutants (E1– E3) were identified from error-prone PCR libraries. The best ethanol-tolerant strain E2 (M59T) had the growth rate of 0.08 h^−1^ in 62 g/L ethanol, higher than that of the control at 0.06 h^−1^. The M59T mutation was then integrated into the genome to create variant iE2. When exposed to 150 g/l ethanol, the survival of iE2 after 15 min was about 12%, while that of BW25113 was <0.01%. Quantitative real-time reverse transcription PCR analysis (RT-PCR) on 444 CRP-regulated genes using OpenArray® technology revealed that 203 genes were differentially expressed in iE2 in the absence of ethanol, whereas 92 displayed differential expression when facing ethanol stress. These genes belong to various functional groups, including central intermediary metabolism (*aceE*, *acnA*, *sdhD*, *sucA*), iron ion transport (*entH*, *entD*, *fecA*, *fecB*), and general stress response (*osmY*, *rpoS*). Six up-regulated and twelve down-regulated common genes were found in both iE2 and E2 under ethanol stress, whereas over one hundred common genes showed differential expression in the absence of ethanol. Based on the RT-PCR results, *entA*, *marA* or *bhsA* was knocked out in iE2 and the resulting strains became more sensitive towards ethanol.

## Introduction

The use of bioethanol as alternative fuel has drawn greater attention than ever due to recent energy crisis and environmental concerns [1], and production of ethanol from microbial fermentation is of practical value in replacing fossil fuel utilization. Different microorganisms, including yeast [2], [3], *Zymomonas mobilis* [4], [5] and *E. coli* [6], [7] have been engineered for selective production of ethanol. The highest reported ethanol yield attained through *E. coli* xylose fermentation is around 60 g/l [8]. Nevertheless, the yields and titers from the microbial fermentation is usually held back by the accumulation of toxic end-product ethanol [9], [10]. As such, it is essential to obtain ethanol-tolerant microbes for large-scale bioethanol production.

In general, there are two conventional approaches to improve strain performance under ethanol stress: i) “random approach” with UV/chemical mutagens [11] and adaptive evolution [8], [12] ii) “rational approach” of using metabolic engineering tools [13], [14]. However, the random introduction of mutations into microbial genetic materials by mutagens is usually time-consuming and laborious. As for the “rational approach”, the lack of detailed metabolism knowledge for many microorganisms often limits its use [15].

An alternative approach in strain engineering, namely transcriptional engineering, has received much attention in recent years. It has been reported before that cell performance can be altered by introducing modifications to transcription factor Spt15 [16], sigma factor [17], zinc-finger containing artificial transcription factor [18], H-NS [19], Hha [20], as well as IrrE [21], [22]. In particular, sigma factor 70 from *E. coli* [23] and IrrE from *Deinococcus radiodurans* had been engineered to improve the ethanol tolerance of *E. coli* DH5α. Our lab has successfully improved the osmotolerance and 1-butanol tolerance of *E. coli* DH5α through engineering its global regulator cAMP receptor protein (CRP) in the past [24]–[27]. In this work, we would like to improve the ethanol tolerance of *E. coli* BW25113 by engineering its CRP. *E. coli* BW25113 is a well-characterized microbe that has been used for gene deletion or chromosomal integration [28]. Both *E. coli* BW25113 and its isogenic mutants have been engineered for the production of chemicals [29]–[31], such as hydrogen [32] and D-lactate [33]. CRP is a well-known *trans*-acting transcription factor that regulates the expression of more than 400 genes in *E. coli* [34]–[37], and participates in various regulatory networks and different metabolic processes [38]–[40]. In view of these discoveries, we speculated that the ethanol tolerance of *E. coli* could also be altered by rewiring its global regulator CRP.

Here, we harnessed directed evolution technique to introduce mutations into CRP [41], and the random mutagenesis libraries were subjected to selection under ethanol stress. Three error-prone PCR variants (E1–E3) with enhanced ethanol resistance were identified. The amino acid substitution in the best ethanol-tolerant mutant E2 was integrated into the genome of *E. coli* JW5702 Δ*kan* to create variant iE2, which was further investigated with respect to its survival and tolerance towards other alcohols. Moreover, changes in the transcript profile of 444 CRP-regulated genes in both iE2 and E2 were examined by quantitative real-time reverse transcription PCR (RT-PCR) using OpenArray**®** real-time PCR technology.

## Materials And Methods

### Materials

*E. coli* JW5702 (BW25113 Δ*crp*) from Keio collection [28] was purchased from *E. coli* Genetic Stock Centre, and used as the host strain for gene manipulation as well as phenotype selection. The kanamycin resistance gene in JW5702 was removed using FLP helper plasmid following protocol to create JW5702 Δ*kan* strain [42]. *E. coli* cells were grown in Luria–Bertani (LB) medium (*w/v*): 0.5% yeast extract (Merck Millipore, Damstadt, Germany), 1% tryptone (Oxoid, Hampshire, UK), and 1% NaCl (Merck Millipore, Damstadt, Germany). Restriction enzymes and *pfu* DNA polymerase were purchased from Fermentas (Burlington, Canada), and T4 DNA ligase was from New England Biolabs (Ipswich, MA, USA). Plasmids pKSC and pKSCP (containing native *crp* operon) were obtained from our previous work [24], [25].

### Random Mutagenesis

Error-prone PCR was performed using Genemorph® II Random Mutagenesis kit (Agilent Technologies, Santa Clara, CA, USA) according to the manufacturer’s instructions. Primers A and B (Table 1) and 30 ng DNA template were used to amplify the full-length *crp* gene. The error-prone PCR program was set as follows: 2 min at 95°C, 30 cycles of 95°C for 1 min, 62°C for 1 min, followed by 72°C for 1 min, and 10 min at 72°C. The PCR products were recovered from 1.0% agarose gel with QIAquick® gel extraction kit (QIAGEN, Germany), digested with restriction enzymes *Bam* HI and *Kpn* I, and inserted into plasmid pKSC that was treated with the same enzymes. The recombinant plasmids constructed were then transformed into *E. coli* JW5702 Δ*kan* by electroporation using an Eppendorf® Multiporator (Hamburg, Germany).

**Table 1:** Primer sequences with restriction site underlined.

Primer	Sequence
A	5′-GAGAGGATCCATAACAGAGGATAACCGCGCATG-3′
B	5′-AGATGGTACCAAACAAAATGGCGCGCTACCAGGTAACGCGCCA-3′
C	5′-GGAAAACATATGATTCCGGGGATCCGTCGACC-3′
D	5′-CGGTATCATATGTTTTCCTGACAGAGTACGCGTACTAACCAAATCG-3′
E	5′-GAATTCGAGCTCGTGTAGGCTGGAGCTGCTTCG-3′
F	5′-GGAAAACATATGATTCCGGGGATCCGTCGACC-3′
G	5′-ATCCGAATTCTGGAAGGAAAGAAAATCGAGTAACTCTGCT-3′
H	5′-CTACACGAGCTCTTGACGCAGTGGAGTAGCAAAAATG-3′
I	5′-TACCCTCGAGCGATGTGGCGCAGACTGATTTATC-3′
J	5′-CCTAGGTTAATTAAGACTTAGTGACTCGCGCAGCGGTTGTTC-3′

### Phenotype Selection

The transformants were cultivated in 50-ml LB medium containing 40 g/L ethanol at 37°C, 200 rpm for 12–16 h, and repeated for two subcultures. Cells were plated onto LB agar plates after each round of selection. Individual colony was randomly picked, and the recombinant plasmids were extracted and sequenced.

### Cell Growth Profile

For each mutant strain, one percent overnight seed (*v/v*) was inoculated into 10-ml fresh LB broth containing various ethanol concentration (0 g/l, and 62 g/l) with a similar initial OD600 value at ∼0.06. Cells were cultured at 37°C with a constant agitation of 200 rpm, and their growth was recorded spectrophotometrically at 600 nm. The *E. coli* JW5702 Δ*kan* harboring pKSCP plasmid was used as control in this study.

### Chromosomal Integration

The *crp* operon from the best CRP mutant was integrated into the chromosome of *E. coli* JW5702 Δ*kan*, the native *crp* operon position, using λ-Red recombination technique [43]. The operon was first amplified using primers C and D (Table 1), while the kanamycin marker (Kan) was amplified from pKD13 using primers E and F. The upstream homology region (H1) was amplified from *E. coli* BW25113 using primer pair G and H, while the downstream homology region (H2) was amplified with primers I and J. All these PCR fragments were ligated into pETDuet-1 (Merck Millipore, Damstadt, Germany) after being digested with appropriate restriction enzymes to produce recombinant plasmid *crp*-HKpETduet. pETDuet-1 was chosen due to its availability of multiple restriction sites. The DNA cassette containing H1-Kan-*crp* operon-H2 was obtained after digesting *crp*-HKpetDuet with *Nde* I and *Pac* I. This DNA cassette was then electroporated into *E. coli* JW5702 Δ*kan* for chromosomal integration, and the site specific localization was verified through PCR. DNA sequencing was performed to confirm that the mutation in the integrated *crp* operon was exactly the same as in the original mutant.

### Survival

The survival of the CRP variant was measured by exposing it to a high ethanol concentration. Both the control and the variant were first cultured in LB medium until their OD600 reached 0.6, followed by the exposure to 150 g/l of ethanol. Aliquots of 10-µl cell culture were taken at an interval of 5 min for a total period of 60 min, and serial dilutions were plated onto LB agar plates containing 30 µg/ml kanamycin. The number of colonies was counted after overnight incubation at 37°C. “Survival” is defined as the ratio of CFU after exposure to ethanol to the CFU when ethanol is absent.

### RNA Isolation

One percent (*v/v*) overnight cell culture was inoculated into LB medium with or without 60 g/l ethanol. Cells were cultured for 2.5 h in the absence of ethanol, or for 8 h in 60 g/l ethanol. The isolation of total RNA was performed using PureLink® RNA mini kit (Life Technologies, Carlsbad, CA, USA), with PureLink® DNase (Life Technologies, Carlsbad, CA, USA) treatment according to the manufacturer’s instructions. The quality and integrity of the isolated RNA was determined through spectrophotometer and agarose gel electrophoresis. About 800 ng total RNA was converted into cDNA by reverse transcription in a 20-µl reaction mixture using High Capacity cDNA Reverse Transcription Kit (Life Technologies, Carlsbad, CA, USA) with random primer mix provided following the recommended protocol.

### Quantitative Real-time Reverse Transcription PCR (RT-PCR) using OpenArray® Technology

RT-PCR using OpenArray® Real-time PCR instrument (Life Technologies, Carlsbad, CA, USA) was performed in triplicates. The OpenArray® technology is a high throughput real-time PCR platform, which allows assessing the expression of 244 genes on one single plate. The Lightcycler® (Roche, Germany) FastStart® (Roche, Germany) DNA Master SYBR® (Life Technologies, Carlsbad, CA, USA) Green I master mix (Roche, Germany) was used in reformatted OpenArray® real-time PCR plates (Life Technologies, Carlsbad, CA, USA). 33-nl reaction mixture was loaded into each through-hole on the OpenArray® real-time PCR plates with the OpenArray® AccuFill™ system (Life Technologies, Carlsbad, CA, USA). The bacterial 16S rRNA (*rrsG*) was used as internal standard and the sequence of the primers are given in Table S1. The values of cycle threshold (C_t_) were provided by the OpenArray® Real-time PCR Analysis Software Version 1.0.4, and 2^−ΔΔCt^ method of relative quantification was utilized to compute the relative expression level. The *p*-value was calculated by student’s *t*-test using IBM SPSS Statistics Software Version 19.

### Cross Resistance to other Alcohols

Cell culture was prepared by diluting overnight seed into fresh LB medium containing different alcohols. Cell growth was recorded by OD600 readings with the incubation at 37°C, 200 rpm. The alcohol concentrations used are presented in volume ratio: 3.1% 1-propanol, 1.3% 1-butanol, and 0.45% 1-pentanol.

## Results

### Isolation of Ethanol-tolerant CRP Mutants

Error-prone PCR was carried out to introduce 2–4 nucleotide substitutions per *crp* by varying the amount of DNA template. Recombinant plasmids with mutated *crp* inserts were transformed into competent *E. coli* JW5702 Δ*kan* (*crp* knock-out strain) and the total error-prone library size was greater than 10^6^. The mutagenesis libraries were then enriched through repeated subcultures containing 40–45 g/l ethanol to separate “winners” with enhanced ethanol tolerance. The mutated *crp* inserts of these “winners” were digested, re-ligated to freshly prepared plasmid pKSC, and the resulting recombinant plasmids were re-transformed into *E. coli* JW5702 Δ*kan* background to eliminate false positives or chromosomal mutations. Three ethanol-tolerant mutants (E1–E3) with improved growth under ethanol stress were selected and their amino acid substitutions are summarized in Table 2.

**Table 2:** Amino acid substitutions in E1–E3.

Mutant	Amino acid substitutions
E1	H31D D53N G177A
E2	M59T
E3	V47E Q80L

### Growth of CRP Mutants

**Figure 1:**
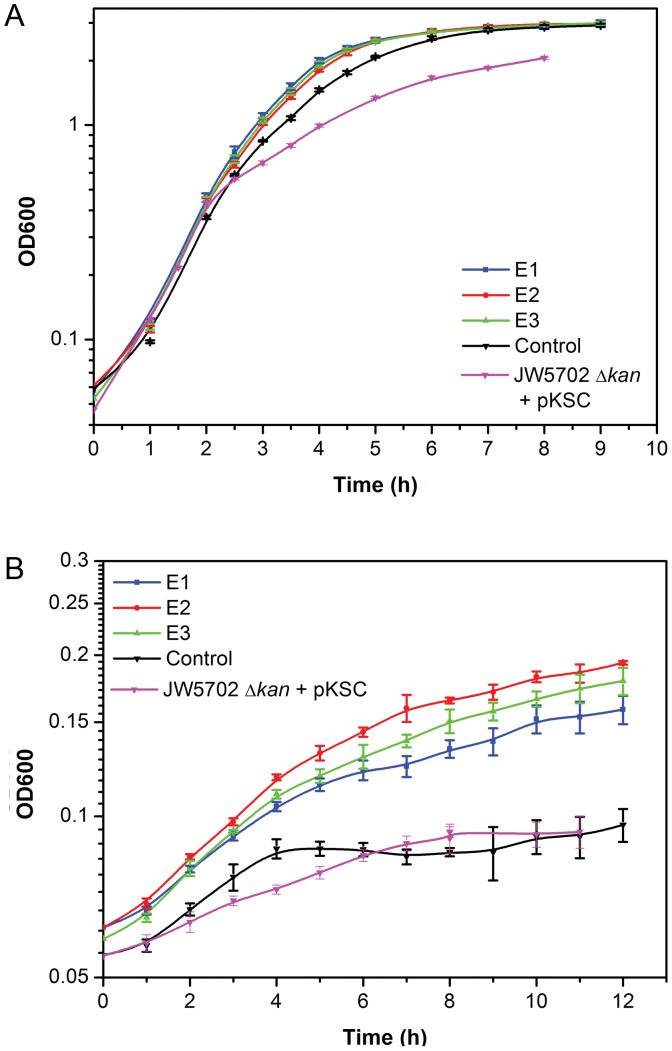
Growth of CRP variants (E1–E3) in LB medium. Cells were grown in (**A**) 0 g/L ethanol (**B**) 62 g/L ethanol at 37°C.

*E. coli* JW5702 Δ*kan* harboring plasmid pKSCP, which contains the native *crp* operon, was used as control in this study. The ethanol tolerance of E1–E3 was investigated at high ethanol concentration (62 g/l) by comparing their growth performance to that of the control and (JW5702 Δ*kan*+blank plasmid pKSC). When cultured without ethanol, all mutants and the control presented similar cell growth rate around 0.48 h^−1^ (Figure 1A). With increasing ethanol concentration in the culture medium, E1–E3 demonstrated better growth than that of the control with E2 displaying the best ethanol tolerance. When ethanol concentration reached 62 g/l (Figure 1B ), the growth rate of E2 was calculated at 0.08 h^−1^, compared to the control’s 0.06 h^−1^. The figure also illustrated a longer exponential growth phase to a higher OD600 value (0.19) than the control (0.10). It was noted that the deletion of *crp* (JW5702 Δ*kan*+blank plasmid pKSC) did not improve the ethanol tolerance of *E. coli.*

### Chromosomal Integration

**Figure 2:**
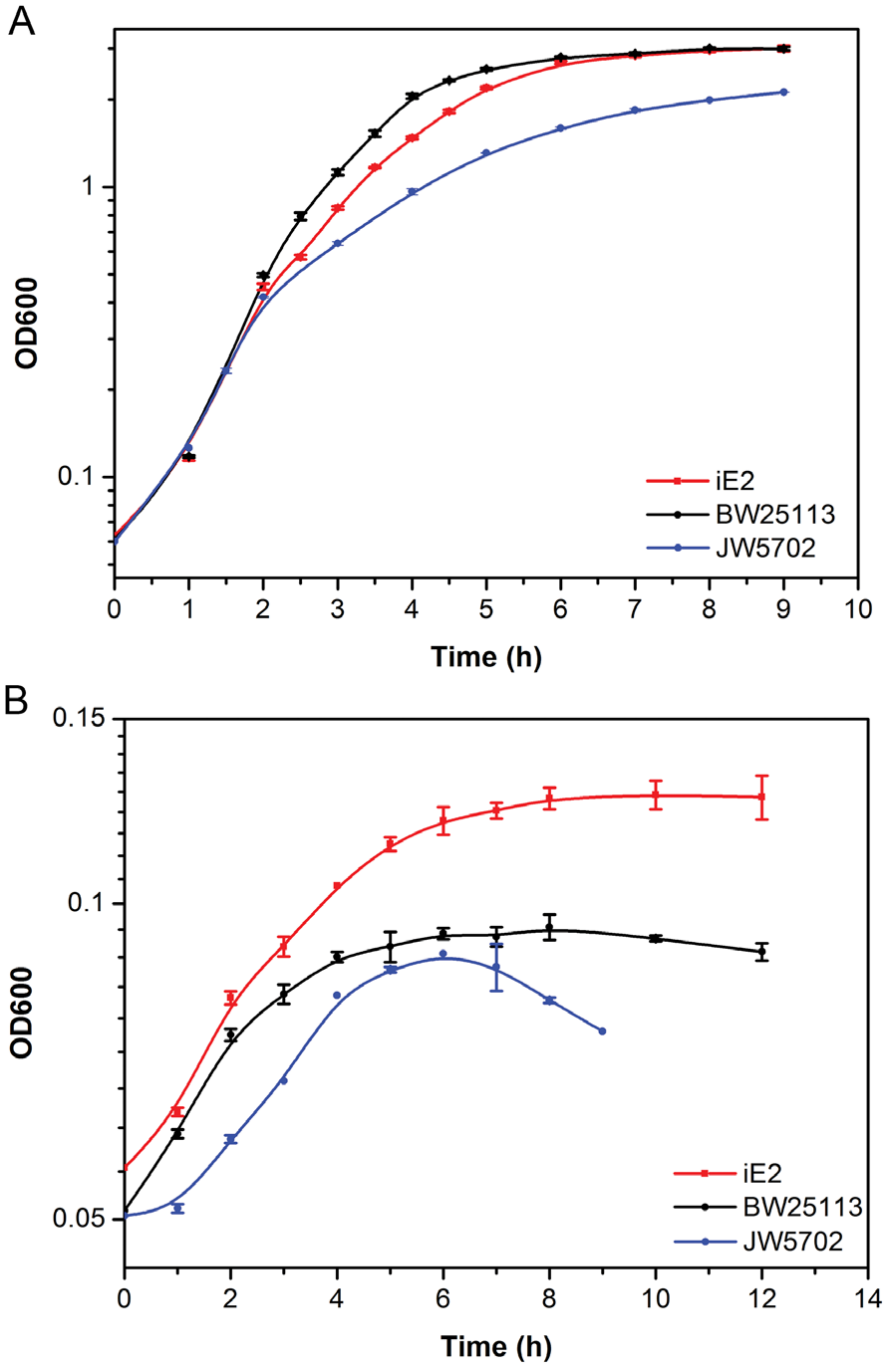
Growth of iE2, parent strain BW25113, and JW5702 in LB medium. Cells were grown in (**A**) no ethanol (**B**) 62 g/L ethanol at 37°C.

Since E2 had demonstrated the best ethanol resistance among all three mutants, its *crp* operon was integrated into the chromosome of *E. coli* JW5702 Δ*kan*, the native *crp* operon location, to create strain iE2. Chromosomal integration was performed to avoid the disadvantages of using plasmid-based system, such as genetic instability arising from segregation or horizontal gene transfer, metabolic burden, and the need for supplementing antibiotics [44], [45]. DNA sequencing results confirmed the same amino acid substitution in the *crp* operon of iE2 as E2. The growth of iE2 under ethanol stress was investigated with its parent strain BW25113 and JW5702. In the absence of ethanol, iE2 shared similar growth pattern with BW25113, with a growth rate around 0.45 h^−1^, faster than that of JW5702 (Figure 2A). When all strains were facing ethanol challenge (62 g/l ethanol), iE2 (0.07 h^−1^) not only outgrew BW25113 (0.055 h^−1^) but also reached a higher OD600 value (0.13) than BW25113 (0.09) after 12 h (Figure 2B). The *crp* knock-out strain JW5702 exhibited the worst ethanol tolerance among all three strains. It was also noted that when ethanol concentration was low, iE2 might result in worse growth than the parent strain (data not shown).

**Figure 3:**
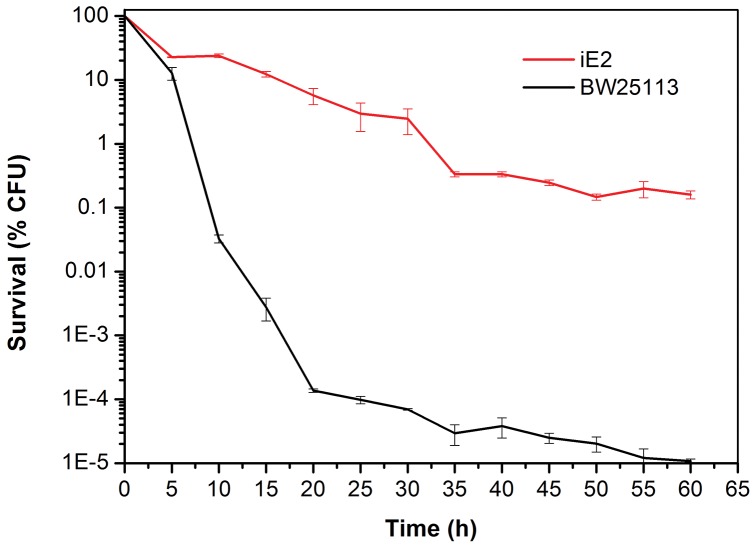
Survival comparison between iE2 and parent strain BW25113 when exposed to 150 g/L ethanol. Cells were grown to mid-exponential phase (OD600 ∼0.6) in LB medium before being exposed to 150 g/L ethanol for a period of 1 h.

To further prove the ethanol tolerance of iE2, both iE2 and BW25113 were exposed to 150 g/l ethanol and their survival was recorded over time (Figure 3). iE2 exhibited significantly better survival than BW25113 over the 1-h period examined. For instance, after 15-min exposure to 150 g/l ethanol, iE2 displayed more than 10% survival whereas BW25113 only had less than 0.01%. Even after 1-h exposure, iE2 still demonstrated over 10,000-fold survival than BW25113.

### Resistance towards other Alcohols

**Figure 4:**
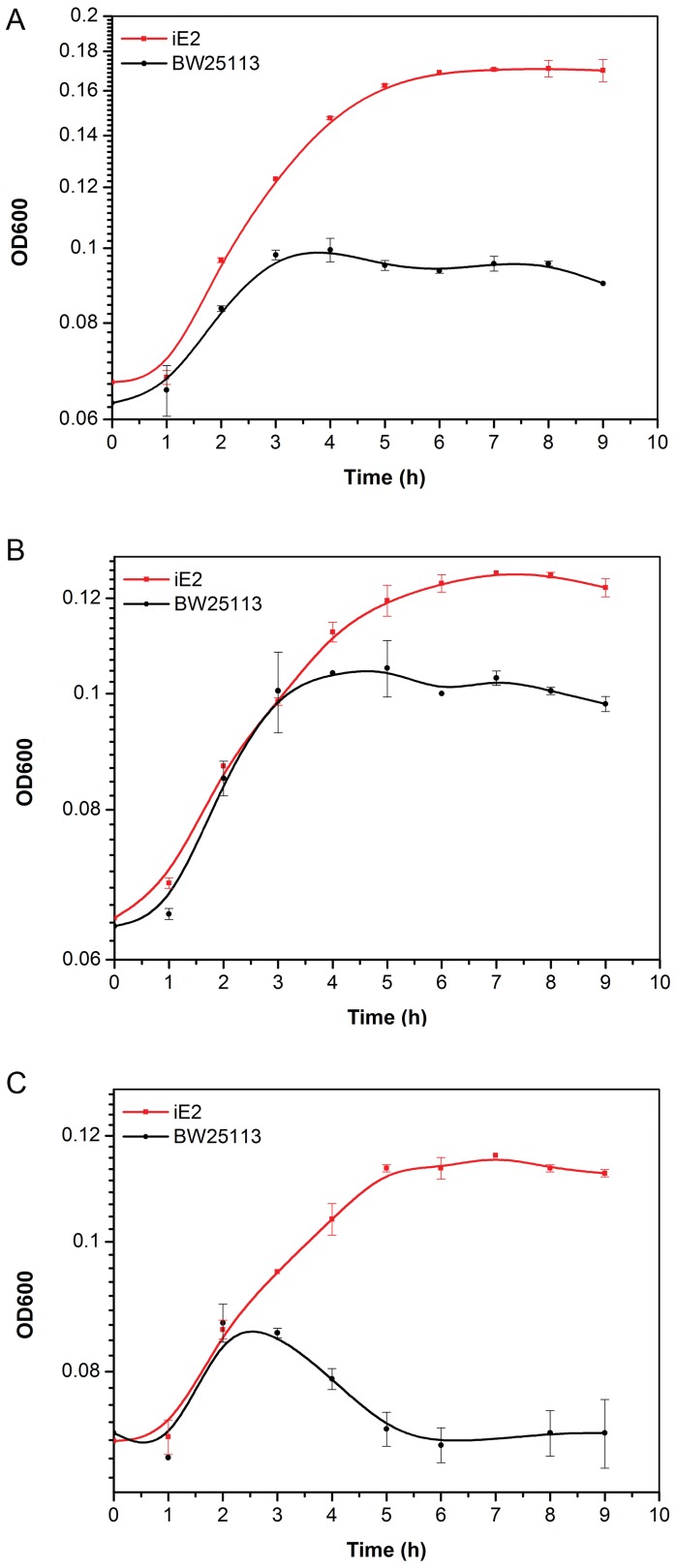
iE2 tolerance towards other alcohols as compared to parent strain BW25113. Cells were cultivated at 37°C in LB medium containing different alcohols: (**A**) 3.1% (*v/v*) 1-propanol, (**B**) 1.3% (*v/v*) 1-butanol, (**C**) 0.45% (*v/v*) 1-pentanol.

The tolerance ability of iE2 towards other alcohols, namely 1-propanol, 1-butanol, and 1-pentanol, was also studied to demonstrate its alcohol tolerance in general (Figure 4). iE2 showed much better growth than BW25113 and was able to achieve a higher OD600 value at stationery phase with all alcohols tested. For instance, the presence of 3.1% (*v/v*) 1-propanol led to 0.129 h^−1^ growth rate in iE2, higher than that of BW25113 at 0.087 h^−1^. Although iE2 and BW25113 shared similar growth rate (0.068 h^−1^) when cultivated in 1.3% (*v/v*) 1-butanol, iE2 was able to reach a higher OD600 value.

### RT-PCR Analysis using OpenArray® System

In order to investigate the transcription level changes of the 444 CRP-regulated genes listed in Ecocyc, we have adopted RT-PCR technique *via* OpenArray® system [34]. It was revealed that 203 genes were differentially expressed in iE2 by more than two-fold with a *p*-value threshold <0.05 in the absence of ethanol, including 48 up-regulated genes and 155 down-regulated genes (Table S2). Genes involved in acid stress (*gadA*, *gadB*, *gadC*) displayed boosted expression (>20-fold), whereas transporter genes, such as *tnaB*, *glpT*, *srlA* and *mglB*, were greatly repressed (>50-fold). Moreover, genes from central intermediary metabolism (*acnA*, *sdhD*, *sucA*) were up-regulated in iE2 by 2–4 folds (Table 3). On the other hand, ethanol stress resulted in the differential expression of 92 genes in iE2 as compared to BW25113, including 71 up-regulated and 21 down-regulated (Table S3), among which many encode for membrane proteins (*yjcH*, *nmpC*, and *osmY*) or transporters (*mdtE*, *fecD*, *ptsG*, *srlA*, and *srlB*) (Table 3).

**Table 3:** Common genes in iE2 or E2 with differential expression in the presence or absence of ethanol stress.

Function		Gene	Fold-change* (iE2/BW25113)		Fold-change* (E2/control)	
			−Ethanol	+Ethanol	−Ethanol	+Ethanol
Transport		*srlA*	0.017	0.028	0.52	0.072
		*srlB*	0.041	0.025	0.38	0.088
		*mdtE*	17.2	5.5	2.8	0.26
		*tnaB*	0.012	4.3	0.039	0.35
		*glpT*	0.014		0.22	0.35
		*mglB*	0.026	2.5	0.030	0.60
		*ptsG*	0.72	0.23	3.3	0.20
Membrane protein		*yjcH*	0.59	8.8	0.13	
		*nmpC*	0.065	3.3		0.38
Enterobactin biosynthesis		*entA*	0.063		0.10	7.5
		*entB*	0.11	1.4	2.05	2.1
		*entE*	0.1	0.61	0.50	3.8
		*entD*	0.078	2.4	–	2.1
Iron transport		*fecA*	0.59	1.7	2.5	0.31
		*fecB*	0.73		1.7	0.75
		*fecC*	0.66	3.9	1.8	
		*fecD*	0.75	4.4		
		*fecE*		2.9	1.4	
Stress response		*gadA*	20.3		2.7	
		*gadB*	50.5		7.2	
		*gadC*	44.8		6.5	
		*rpoS*	2.1	1.3	0.67	
		*osmY*	18.7	2.2	1.5	
		*marA*	1.3	3.1	0.48	2.7
		*bhsA*	3.5	2.5	0.41	4.1
TCA cycle		*acnA*	3.2	0.66	1.2	1.3
		*sdhD*	2.3		0.37	0.74
		*sucA*	2.0		1.6	
Amino acid metabolism		*ilvN*	0.11	1.7	0.3	0.13
Energy metabolism		*fixA*				27.4
		*tdcE*	0.17	11.2	0.25	0.33
Carbon compound catabolism		*galE*	0.10	22.3	0.001	265
		*gatD*	0.15		0.3	0.11

* Fold-changes with *p*>0.05 are not shown in this table.

We have also investigated on the transcription changes of these 444 genes in E2 for comparison. A total of 169 genes (34 up-regulated and 135 down-regulated) showed differential expression in E2 in the absence of ethanol (Table S4), whereas 159 genes, including 28 up-regulated and 131 down-regulated, were expressed differentially in E2 with ethanol stress present (Table S5). By comparing the differentially expressed genes from iE2 and E2, it was found that 14 up-regulated and 93 down-regulated genes were common in both microbes when treated without ethanol (Table S6). When challenged by ethanol, only 6 up-regulated and 12 down-regulated genes were common in both strains (Table S7). Majority of these commonly down-regulated genes were transport-related, including genes encoding for glucitol/sorbitol sugar transporter of the phosphotransferase system (PTS), namely *srlA*, *srlB*, *srlD* and *srlE*, which were highly down-regulated in both iE2 (16- to 40- fold) and E2 (7- to 14-fold).

## Discussion

In the present study, we have used transcriptional engineering tools to enhance the ethanol tolerance of *E. coli* by rewiring its global regulator CRP. Mutations were introduced to CRP *via* error-prone PCR and three variants were identified with enhanced ethanol tolerance. The *crp* operon from the best error-prone PCR mutant had been integrated into genome to create mutant strain iE2, which also demonstrated better tolerance than its parent *E. coli* strain BW25113 at high ethanol concentration.

CRP is composed of an N-terminal cAMP binding domain (residues 1–134) and a C-terminal DNA recognition and binding domain (residues 140–209) that are connected by a hinge region (residues 135–139) [46]. The M59T mutation from E2 is located in β-strand 5, which is known to be involved in the interdomain contacts of CRP-cAMP complex [47]. Saturated mutagenesis at location M59 didn’t generate a much improved ethanol-tolerant variant (data not shown).

Ethanol-tolerant strains can be attained *via* genetic modifications by altering their membrane structural components or reprograming metabolic processes [48]. Contrary to previous findings whereby elevated gene expression of tricarboxylic acid (TCA) cycle was found in ethanol-tolerant *E. coli* strain [12], [48], [49], the fold-change for the majority genes in the TCA cycle in iE2 or E2 was either having less than two-fold change or the change was statistically insignificant (*p*>0.05) with or without ethanol treatment. iE2 had also demonstrated an elevated expression in genes involved in general stress response (*rpoS* and *osmY*) and drug resistance (*marA*), which was in agreement with previous reports on the transcriptome analysis of ethanol tolerant *E. coli* strain LY01 obtained *via* spontaneous adaptation [49]. Genes involved in iron ion transport (*fecA*, *fecB*, *fecC*, *fecD* and *fecE*) and enterobactin biosynthesis pathway (*entA*, *entB*, *entC*, *entD*, *entE* and *entH*) were also up-regulated in this ethanol tolerant strain [12], [48]. Likewise, we have also found the expression level of *fecC*, *fecD* and *fecE* and *entD* were higher in iE2 under ethanol stress (2.4- to 4.4-fold). Besides *entD*, other enterobactin biosynthesis genes such as *entA*, *entB* and *entE* were also enhanced in mutant strain E2.

Among the commonly up-regulated genes from iE2 and E2, *bhsA* and *marA* are of particular interest. It was previously reported that *bhsA*, encoding putative outer membrane protein, could be induced by several stress conditions and its deletion would result in acid, heat, hydrogen peroxide or cadmium sensitivity [50]–[53], while elevated gene expression of *marA*, encoding multiple antibiotics resistance protein, was observed in ethanol-tolerant strain LY01. Despite the fact that *marA* was up-regulated in iE2, E2, and LY01 under ethanol stress, the overexpression of *marA* didn’t improve the ethanol tolerance of *E. coli* [49].

Based on the RT-PCR results, we have attempted to overexpress ten genes in *E. coli* BW25113, including genes encoding transporters (*mdtE*, *srlA*, and *srlB*), membrane protein (*yjcH*), and enzymes (*entA*, *tdcE*, *galE*, *gatD*, *ilvN*, *fixA*), but neither improvement nor decline in ethanol tolerance were discovered following their overexpression (data not shown). One possible reason could be that ethanol tolerance is elicited through the involvement of multiple pathways in *E. coli*, and single genetic modification is not sufficient to lead to substantial increase in ethanol tolerance [48], [54].

After comparing the RT-PCR data between iE2 and E2, we found that *marA* and *bhsA* were up-regulated under ethanol stress, and both were reported previously to play certain roles in stress resistance [50], [55]. Furthermore, as mentioned earlier, *entA*, only up-regulated in E2, was also related with ethanol tolerance. Hence, we knocked out *entA*, *marA* and *bhsA* from iE2 to investigate on their effect on iE2 ethanol tolerance. As shown in Figure S1, it was evident that iE2 Δ*entA*, iE2 Δ*marA* and iE2 Δ*bhsA* not only had a lower growth than iE2, but also attained lesser OD600 values at stationary phase. Therefore, iE2 is likely to increase its ethanol tolerance through *entA*, *marA* and *bhsA*.

Conventional strain engineering tools were adopted in the past to select for ethanol-tolerant *E. coli*. For example, the ethanol tolerance of *E. coli* was enhanced by either spontaneous adaptation on medium supplemented with ethanol [8], [13] or metabolic engineering method [14]. However, these approaches are often resource-intensive, for instance, adaptive evolution method requires a culturing period of ∼1000 h to obtain an enhanced ethanol-tolerant strain [12], or require specific genetic and metabolic information. As for using UV/chemical mutagens to improve cellular functions, mutations are often difficult to map in the genome, which makes the improvement mechanism inaccessible for new strain engineering [56]. Strain engineering *via* global regulator CRP, on the other hand, was able to generate several enhanced mutant strains in a couple of days. Moreover, the information obtained from one cell phenotype improvement may also help guide phenotype improvement under other stresses due to the overlap of cell stress response. Based on the results in this study and our previous findings, we believe that transcriptional engineering through CRP can offer an efficient and convenient approach for strain engineering.

## Supporting Information

Figure S1 **Growth profiles of iE2 and its knockout strains.** Cells were grown in (A) 0 g/l, and (B) 55 g/l ethanol.
(TIF)

Table S1 Primers used in OpenArray® real-time PCR.
(DOCX)

Table S2 Genes with >2-fold change in their expression level in iE2 as compared to BW25113 in the absence of ethanol, using a *p*-value threshold less than 0.05.
(DOCX)

Table S3 Genes with >2-fold change in their expression level in iE2 as compared to BW25113 in the prence of ethanol stress, using a *p*-value threshold less than 0.05.
(DOCX)

Table S4 Genes with >2-fold change in their expression level in E2 as compared to the control in the absence of ethanol stress, using a *p*-value threshold less than 0.05.
(DOCX)

Table S5 Genes with >2-fold change in their expression level in E2 as compared to the control in the presence of ethanol stress, using a *p*-value threshold less than 0.05.
(DOCX)

Table S6 Common genes that were either up-regulated OR down-regulated in both iE2 and E2 as compared to their controls in the absence of ethanol stress.
(DOCX)

Table S7 Common genes that were either up-regulated OR down-regulated in both iE2 and E2 as compared to their controls in the presence of ethanol stress.
(DOCX)
