# Supplementary figures and images for: Improving Ethanol Tolerance of Escherichia coli by Rewiring Its Global Regulator cAMP Receptor Protein (CRP)
Source: PLoS One. 2013 Feb 28;8(2):e57628. doi: 10.1371/journal.pone.0057628 (PMC3585226; doi:10.1371/journal.pone.0057628)

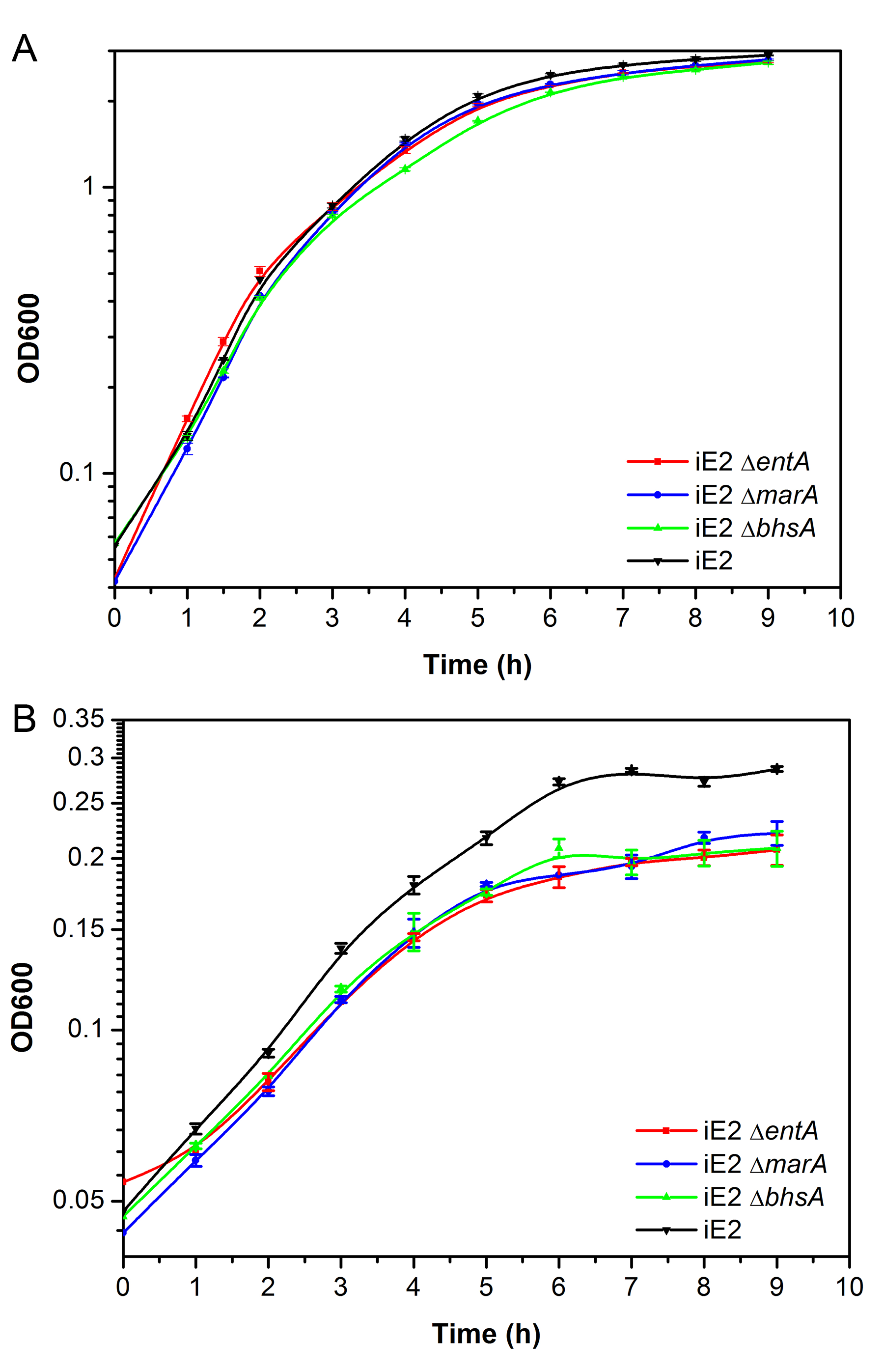

Supplement: Figure S1 — Growth profiles of iE2 and its knockout strains. Cells were grown in (A) 0 g/l, and (B) 55 g/l ethanol. (TIF) [file pone.0057628.s001.tif]
