# Supplementary material for: Improving Ethanol Tolerance of Escherichia coli by Rewiring Its Global Regulator cAMP Receptor Protein (CRP)
Source: PLoS One. 2013 Feb 28;8(2):e57628. doi: 10.1371/journal.pone.0057628 (PMC3585226; doi:10.1371/journal.pone.0057628)
Supplement: Table S2 — Genes with >2-fold change in their expression level in iE2 as compared to BW25113 in the absence of ethanol, using a p-value threshold less than 0.05. (DOCX) [file pone.0057628.s003.docx]

**TABLE S2**. Genes with >2-fold change in their expression level in iE2 as compared to BW25113 in the absence of ethanol, using a *p*-value threshold less than 0.05.

| **b-number** | **Gene** | **Function^a^** | **Fold-change^b^** | ***p*-value** |
| --- | --- | --- | --- | --- |
| b1493 | *gadB* | glutamate decarboxylase B | 50.525 | 6.81E-04 |
| b1492 | *gadC* | GadC GABA APC transporter | 44.773 | 6.35E-05 |
| b3517 | *gadA* | glutamate decarboxylase A | 20.320 | 3.63E-06 |
| b4376 | *osmY* | periplasmic protein | 18.726 | 1.38E-07 |
| b3512 | *gadE* | GadE DNA-binding transcriptional activator | 18.309 | 4.45E-07 |
| b3513 | *mdtE* | MdtEF-TolC multidrug efflux transport system - membrane fusion protein | 17.156 | 2.92E-07 |
| b3514 | *mdtF* | MdtEF-TolC multidrug efflux transport system - permease subunit | 7.786 | 1.64E-07 |
| b1182 | *hlyE* | hemolysin E | 7.288 | 9.26E-03 |
| b3516 | *gadX* | GadX DNA-binding transcriptional dual regulator | 7.209 | 6.57E-07 |
| b4030 | *psiE* | predicted phosphate starvation-inducible protein | 5.844 | 2.79E-08 |
| b4111 | *proP* | ProP osmosensory MFS transporter | 5.748 | 4.11E-07 |
| b3453 | *ugpB* | glycerol-3-phosphate / glycerol-2-phosphate ABC transporter - putative periplasmic binding protein | 5.276 | 3.22E-06 |
| b2508 | *guaB* | IMP dehydrogenase | 5.066 | 2.93E-04 |
| b2661 | *gabD* | succinate semialdehyde dehydrogenase, NADP+-dependent | 5.064 | 6.84E-07 |
| b2659 | *csiD* | predicted protein | 5.040 | 1.11E-02 |
| b1519 | *tam* | trans-aconitate methyltransferase | 4.769 | 4.71E-05 |
| b0343 | *lacY* | LacY lactose MFS transporter | 4.412 | 3.48E-02 |
| b3449 | *ugpQ* | glycerophosphodiester phosphodiesterase, cytosolic | 4.266 | 1.70E-08 |
| b4014 | *aceB* | malate synthase A | 4.008 | 9.09E-07 |
| b3450 | *ugpC* | glycerol-3-phosphate / glycerol-2-phosphate ABC transporter - putative ATP binding subunit | 3.555 | 8.84E-04 |
| b2507 | *guaA* | GMP synthetase | 3.551 | 3.63E-05 |
| b0124 | *gcd* | glucose dehydrogenase | 3.541 | 4.13E-03 |
| b1112 | *bhsA* | protein involved in stress resistance and biofilm formation | 3.488 | 2.76E-05 |
| b1276 | *acnA* | aconitate hydratase 1 | 3.177 | 2.47E-09 |
| b1038 | *csgF* | curli assembly component | 3.163 | 3.26E-03 |
| b0342 | *lacA* | galactoside O-acetyltransferase | 3.037 | 2.86E-02 |
| b3172 | *argG* | argininosuccinate synthase | 2.811 | 2.74E-08 |
| b2234 | *nrdA* | ribonucleoside diphosphate reductase 1, α subunit dimer | 2.653 | 1.94E-02 |
| b3748 | *rbsD* | ribose pyranase | 2.614 | 1.05E-05 |
| b3437 | *gntK* | D-gluconate kinase, thermostable | 2.598 | 9.57E-07 |
| b0346 | *mhpR* | MhpR transcriptional activator | 2.522 | 1.82E-07 |
| b1272 | *sohB* | predicted inner membrane peptidase | 2.513 | 2.78E-07 |
| b4016 | *aceK* | isocitrate dehydrogenase phosphatase / isocitrate dehydrogenase kinase | 2.499 | 2.63E-05 |
| b3749 | *rbsA* | ribose ABC transporter - putative ATP binding subunit | 2.477 | 8.66E-07 |
| b2235 | *nrdB* | ribonucleoside diphosphate reductase 1, β subunit dimer | 2.474 | 4.38E-05 |
| b4015 | *aceA* | isocitrate lyase | 2.455 | 6.59E-07 |
| b2660 | *lhgO* | L-2-hydroxyglutarate oxidase | 2.444 | 2.52E-03 |
| b2487 | *hyfG* | hydrogenase 4, large subunit | 2.381 | 5.99E-04 |
| b0722 | *sdhD* | succinate dehydrogenase membrane protein | 2.268 | 3.92E-04 |
| b0907 | *serC* | phosphohydroxythreonine aminotransferase / 3-phosphoserine aminotransferase | 2.231 | 4.39E-05 |
| b0432 | *cyoA* | cytochrome bo terminal oxidase subunit II | 2.187 | 7.83E-06 |
| b3451 | *ugpE* | glycerol-3-phosphate / glycerol-2-phosphate ABC transporter - putative membrane subunit | 2.133 | 6.01E-06 |
| b0430 | *cyoC* | cytochrome bo terminal oxidase subunit III | 2.118 | 2.42E-07 |
| b0819 | *ybiS* | L,D-transpeptidase YbiS | 2.116 | 8.18E-05 |
| b2417 | *crr* | glucose-specific enzyme IIA component of PTS | 2.063 | 2.02E-03 |
| b2965 | *speC* | ornithine decarboxylase, biosynthetic | 2.058 | 2.84E-05 |
| b2741 | *rpoS* | RNA polymerase, sigma S (sigma 38) factor | 2.050 | 1.10E-05 |
| b0726 | *sucA* | 2-oxoglutarate decarboxylase, thiamin-requiring | 2.011 | 4.83E-05 |
| b4067 | *actP* | acetate / glycolate transporter | 0.498 | 2.43E-02 |
| b2492 | *focB* | FocB formate FNT transporter | 0.490 | 3.96E-04 |
| b4197 | *ulaE* | L-xylulose 5-phosphate 3-epimerase | 0.484 | 1.04E-02 |
| b2805 | *fucR* | FucR transcriptional activator | 0.482 | 1.42E-07 |
| b4195 | *ulaC* | L-ascorbate-specific enzyme IIA component of PTS | 0.482 | 1.90E-03 |
| b1620 | *malI* | MalI DNA-binding transcriptional repressor | 0.467 | 2.36E-07 |
| b3414 | *nfuA* | iron-sulfur cluster scaffold protein | 0.450 | 6.07E-08 |
| b0678 | *nagB* | glucosamine-6-phosphate deaminase | 0.442 | 3.74E-06 |
| b3905 | *rhaS* | RhaS transcriptional activator | 0.437 | 1.60E-03 |
| b4194 | *ulaB* | L-ascorbate-specific enzyme IIB component of PTS | 0.436 | 1.15E-03 |
| b1819 | *manZ* | mannose PTS permease - ManZ subunit | 0.430 | 7.05E-07 |
| b0347 | *mhpA* | 3-(3-hydroxyphenyl)propanoate hydroxylase | 0.421 | 1.51E-02 |
| b4138 | *dcuA* | DcuA dicarboxylate Dcu transporter | 0.415 | 1.50E-04 |
| b0113 | *pdhR* | PdhR DNA-binding transcriptional dual regulator | 0.414 | 1.89E-06 |
| b4196 | *ulaD* | 3-keto-L-gulonate 6-phosphate decarboxylase | 0.404 | 1.79E-03 |
| b2468 | *aegA* | putative oxidoreductase, Fe-S subunit | 0.400 | 2.02E-04 |
| b3418 | *malT* | MalT transcriptional activator | 0.399 | 3.53E-07 |
| b4213 | *cpdB* | 2',3'-cyclic nucleotide 2'-phosphodiesterase / 3'-nucleotidase | 0.398 | 8.66E-08 |
| b0903 | *pflB* | pyruvate formate-lyase (inactive) | 0.396 | 4.66E-06 |
| b3236 | *mdh* | malate dehydrogenase | 0.390 | 7.65E-05 |
| b1621 | *malX* | fused maltose and glucose-specific PTS enzymes: IIB component, IIC component | 0.386 | 1.69E-03 |
| b2799 | *fucO* | L-1,2-propanediol oxidoreductase | 0.384 | 4.27E-05 |
| b2708 | *gutQ* | D-arabinose 5-phosphate isomerase | 0.384 | 3.29E-05 |
| b1594 | *dgsA* | DgsA DNA-binding transcriptional repressor | 0.370 | 1.61E-03 |
| b2428 | *murQ* | N-acetylmuramic acid 6-phosphate etherase | 0.370 | 3.73E-09 |
| b1389 | *paaB* | ring 1,2-phenylacetyl-CoA epoxidase subunit | 0.369 | 3.13E-04 |
| b3869 | *glnL* | NtrB sensory histidine kinase | 0.366 | 1.68E-07 |
| b3723 | *bglG* | BglG transcriptional antiterminator | 0.361 | 8.59E-05 |
| b1205 | *ychH* | stress-induced protein | 0.360 | 1.79E-05 |
| b3934 | *cytR* | CytR DNA-binding transcriptional repressor | 0.359 | 1.51E-10 |
| b2147 | *preA* | NADH-dependent dihydropyrimidine dehydrogenase subunit | 0.352 | 2.51E-06 |
| b3368 | *cysG* | uroporphyrin III C-methyltransferase [multifunctional] | 0.351 | 1.71E-07 |
| b3868 | *glnG* | NtrC transcriptional dual regulator | 0.350 | 9.74E-04 |
| b0757 | *galK* | galactokinase | 0.343 | 1.52E-05 |
| b1415 | *aldA* | aldehyde dehydrogenase A, NAD-linked | 0.339 | 1.90E-04 |
| b4003 | *zraS* | ZraS sensory histidine kinase | 0.335 | 6.98E-06 |
| b3566 | *xylF* | xylose ABC transporter - periplasmic binding protein | 0.330 | 3.23E-02 |
| b1002 | *agp* | 3-phytase / glucose-1-phosphatase | 0.323 | 2.66E-09 |
| b2344 | *fadL* | long-chain fatty acid outer membrane transporter; sensitivity to phage T2 | 0.320 | 8.21E-09 |
| b2801 | *fucP* | FucP fucose MFS transporter | 0.313 | 4.44E-03 |
| b2146 | *preT* | NADH-dependent dihydropyrimidine dehydrogenase subunit | 0.310 | 3.13E-07 |
| b3925 | *glpX* | fructose 1,6-bisphosphatase II | 0.309 | 3.05E-03 |
| b2964 | *nupG* | NupG nucleoside MFS transporter | 0.305 | 9.96E-10 |
| b4268 | *idnK* | D-gluconate kinase, thermosensitive | 0.298 | 1.37E-03 |
| b4460 | *araH* | arabinose ABC transporter - membrane subunit | 0.285 | 8.03E-07 |
| b4471 | *tdcG* | L-serine deaminase III | 0.284 | 2.87E-03 |
| b3135 | *agaA* | predicted truncated N-acetylgalactosamine-6-phosphate deacetylase | 0.283 | 7.24E-06 |
| b3091 | *uxaA* | D-altronate dehydratase | 0.281 | 7.50E-08 |
| b3113 | *tdcF* | predicted L-PSP (mRNA) endoribonuclease | 0.275 | 6.85E-05 |
| b2803 | *fucK* | L-fuculokinase | 0.274 | 2.02E-07 |
| b3579 | *yiaO* | L-dehydroascorbate transporter, periplasmic binding protein | 0.271 | 1.75E-03 |
| b1392 | *paaE* | ring 1,2-phenylacetyl-CoA epoxidase, reductase subunit | 0.271 | 1.73E-05 |
| b2614 | *grpE* | phage lambda replication; host DNA synthesis; heat shock protein; protein repair | 0.271 | 3.37E-06 |
| b3221 | *yhcH* | conserved protein | 0.268 | 3.54E-10 |
| b4266 | *idnO* | 5-keto-D-gluconate 5-reductase | 0.267 | 3.94E-05 |
| b3092 | *uxaC* | D-glucuronate isomerase / D-galacturonate isomerase | 0.262 | 1.50E-05 |
| b1391 | *paaD* | phenylacetate degradation protein | 0.254 | 1.10E-04 |
| b3565 | *xylA* | xylose isomerase | 0.253 | 8.61E-06 |
| b1521 | *uxaB* | altronate oxidoreductase | 0.249 | 8.87E-06 |
| b0679 | *nagE* | N-acetylglucosamine PTS permease | 0.243 | 2.40E-07 |
| b4323 | *uxuB* | D-mannonate oxidoreductase | 0.239 | 2.21E-05 |
| b4069 | *acs* | acetyl-CoA synthetase (AMP-forming) | 0.236 | 6.09E-06 |
| b2365 | *dsdX* | DsdX Gnt tranporter | 0.234 | 6.24E-08 |
| b1593 | *ynfK* | predicted dethiobiotin synthetase | 0.231 | 1.67E-12 |
| b1818 | *manY* | mannose PTS permease - ManY subunit | 0.228 | 5.05E-11 |
| b1817 | *manX* | mannose PTS permease - ManX subunit | 0.220 | 6.82E-11 |
| b1514 | *lsrC* | AI-2 ABC transporter - membrane subunit | 0.219 | 4.76E-05 |
| b3134 | *agaW* | PTS system N-acetylgalactosameine-specific IIC component 2 | 0.218 | 2.63E-03 |
| b3133 | *agaV* | PTS system, cytoplasmic, N-acetylgalactosamine-specific IIB component 2 (EIIB-AGA) | 0.215 | 1.30E-05 |
| b3870 | *glnA* | glutamine synthetase | 0.213 | 2.96E-12 |
| b3578 | *yiaN* | L-dehydroascorbate transporter | 0.204 | 5.71E-03 |
| b3575 | *yiaK* | 2,3-diketo-L-gulonate reductase | 0.202 | 9.19E-03 |
| b2707 | *srlR* | GutR DNA-binding transcriptional repressor | 0.202 | 2.49E-06 |
| b2841 | *araE* | AraE arabinose MFS transporter | 0.198 | 2.04E-05 |
| b4322 | *uxuA* | D-mannonate dehydratase | 0.196 | 4.27E-11 |
| b2095 | *gatZ* | D-tagatose 1,6-bisphosphate aldolase 2, subunit | 0.194 | 7.45E-05 |
| b0411 | *tsx* | nucleoside channel; receptor of phage T6 and colicin K | 0.184 | 5.66E-12 |
| b2802 | *fucI* | L-fucose isomerase | 0.180 | 2.25E-05 |
| b3132 | *kbaZ* | tagatose 6-phosphate aldolase 1, kbaZ subunit | 0.171 | 1.14E-04 |
| b3114 | *tdcE* | 2-ketobutyrate formate-lyase/pyruvate formate-lyase 4, inactive | 0.167 | 1.44E-05 |
| b4311 | *nanC* | N-acetylneuraminic acid outer membrane channel | 0.166 | 8.06E-05 |
| b2092 | *gatC* | galactitol-specific enzyme IIC component of PTS | 0.164 | 1.26E-09 |
| b3528 | *dctA* | DctA dicarboxylate DAACS transporter | 0.162 | 2.08E-05 |
| b0598 | *cstA* | peptide transporter induced by carbon starvation | 0.161 | 1.20E-03 |
| b2096 | *gatY* | D-tagatose 1,6-bisphosphate aldolase 2, catalytic subunit | 0.159 | 1.28E-04 |
| b1617 | *uidA* | β-D-glucuronidase | 0.155 | 2.91E-07 |
| b2094 | *gatA* | galactitol-specific enzyme IIA component of PTS | 0.151 | 6.45E-09 |
| b0597 | *entH* | proofreading thioesterase in enterobactin biosynthesis | 0.149 | 5.05E-05 |
| b2091 | *gatD* | galactitol-1-phosphate dehydrogenase | 0.147 | 3.16E-08 |
| b4118 | *melR* | MelR DNA-binding transcriptional dual regulator | 0.144 | 1.66E-05 |
| b2366 | *dsdA* | D-serine deaminase | 0.138 | 7.60E-11 |
| b0584 | *fepA* | FepA, outer membrane receptor for ferric enterobactin (enterochelin) and colicins B and D | 0.136 | 1.39E-03 |
| b2093 | *gatB* | galactitol-specific enzyme IIB component of PTS | 0.136 | 2.96E-14 |
| b2579 | *yfiD* | stress-induced alternate pyruvate formate-lyase subunit | 0.136 | 1.81E-07 |
| b0904 | *focA* | FocA formate transporter | 0.129 | 4.25E-07 |
| b2151 | *galS* | GalS DNA-binding transcriptional dual regulator | 0.127 | 7.05E-05 |
| b3927 | *glpF* | GlpF glycerol MIP channel | 0.123 | 8.58E-07 |
| b0595 | *entB* | apo-EntB multimer | 0.115 | 2.43E-05 |
| b3576 | *yiaL* | conserved protein | 0.115 | 5.25E-04 |
| b3670 | *ilvN* | acetolactate synthase I, small subunit | 0.114 | 1.43E-06 |
| b2243 | *glpC* | glycerol-3-phosphate dehydrogenase (anaerobic), small subunit | 0.112 | 7.69E-09 |
| b0908 | *aroA* | 3-phosphoshikimate-1-carboxyvinyltransferase | 0.109 | 3.90E-06 |
| b4321 | *gntP* | GntP Gluconate Gnt transporter | 0.105 | 6.02E-07 |
| b3077 | *ebgC* | evolved β-D-galactosidase, β subunit | 0.103 | 4.43E-06 |
| b3222 | *nanK* | N-acetylmannosamine kinase | 0.102 | 9.61E-06 |
| b2957 | *ansB* | asparaginase II | 0.101 | 2.15E-17 |
| b0759 | *galE* | UDP-glucose 4-epimerase | 0.099 | 4.53E-05 |
| b3581 | *sgbH* | 3-keto-L-gulonate 6-phosphate decarboxylase | 0.099 | 6.96E-03 |
| b3926 | *glpK* | glycerol kinase | 0.098 | 1.03E-04 |
| b0594 | *entE* | 2,3-dihydroxybenzoate-holo-EntB ligase [multifunctional] | 0.096 | 2.09E-05 |
| b4267 | *idnD* | L-idonate 5-dehydrogenase | 0.093 | 4.17E-07 |
| b3671 | *ilvB* | acetolactate synthase I, large subunit | 0.079 | 3.72E-06 |
| b0593 | *entC* | isochorismate synthase 1 | 0.078 | 1.55E-04 |
| b0583 | *entD* | phosphopantetheinyl transferase | 0.078 | 4.37E-06 |
| b3115 | *tdcD* | propionate kinase | 0.077 | 7.24E-04 |
| b2706 | *gutM* | GutM DNA-binding transcriptional activator | 0.076 | 1.72E-05 |
| b4032 | *malG* | maltose ABC transporter - membrane subunit | 0.076 | 8.94E-07 |
| b3116 | *tdcC* | TdcC threonine STP transporter | 0.074 | 1.08E-07 |
| b3076 | *ebgA* | evolved β-D-galactosidase, α subunit | 0.071 | 4.93E-08 |
| b0929 | *ompF* | outer membrane porin F | 0.071 | 6.00E-10 |
| b4239 | *treC* | trehalose-6-phosphate hydrolase | 0.069 | 2.93E-05 |
| b0553 | *nmpC* | outer membrane porin protein; locus of qsr prophage | 0.065 | 6.29E-07 |
| b0596 | *entA* | 2,3-dihydro-2,3-dihydroxybenzoate dehydrogenase | 0.063 | 2.54E-05 |
| b2705 | *srlD* | sorbitol-6-phosphate dehydrogenase | 0.060 | 4.49E-06 |
| b1900 | *araG* | arabinose ABC transporter - ATP binding subunit | 0.056 | 1.62E-02 |
| b4240 | *treB* | fused trehalose(maltose)-specific PTS enzyme: IIB component/IIC component | 0.054 | 1.54E-04 |
| b4033 | *malF* | maltose ABC transporter - membrane subunit | 0.052 | 2.27E-13 |
| b0805 | *fiu* | putative outer membrane receptor for iron transport | 0.052 | 3.62E-06 |
| b2155 | *cirA* | outer membrane receptor involved in uptake of ferric dihyroxybenzoylserine | 0.051 | 3.68E-08 |
| b3224 | *nanT* | NanT sialic acid MFS transporter | 0.050 | 1.53E-04 |
| b1901 | *araF* | arabinose ABC transporter - periplasmic binding protein | 0.049 | 5.32E-07 |
| b3367 | *nirC* | NirC nitrite FNT transporter | 0.048 | 1.54E-05 |
| b4037 | *malM* | maltose regulon periplasmic protein | 0.042 | 5.35E-07 |
| b2704 | *srlB* | glucitol/sorbitol-specific enzyme IIA component of PTS | 0.041 | 2.10E-07 |
| b4139 | *aspA* | aspartate ammonia-lyase | 0.037 | 3.49E-06 |
| b2148 | *mglC* | galactose ABC transporter - membrane subunit | 0.036 | 6.34E-07 |
| b4034 | *malE* | maltose ABC transporter - periplasmic binding protein | 0.034 | 1.85E-05 |
| b2242 | *glpB* | glycerol-3-phosphate dehydrogenase (anaerobic), membrane anchor subunit | 0.034 | 5.07E-06 |
| b2149 | *mglA* | galactose ABC transporter - ATP binding subunit | 0.032 | 2.51E-06 |
| b3365 | *nirB* | dimer of large subunit of nitrite reductase | 0.030 | 1.31E-10 |
| b3118 | *tdcA* | TdcA DNA-binding transcriptional activator | 0.030 | 1.13E-04 |
| b3366 | *nirD* | nitrite reductase, small subunit | 0.028 | 5.57E-07 |
| b2703 | *srlE* | glucitol/sorbitol-specific enzyme IIB component of PTS | 0.027 | 2.23E-06 |
| b4036 | *lamB* | phage lambda receptor protein; maltose high-affinity receptor | 0.026 | 3.90E-06 |
| b2150 | *mglB* | galactose ABC transporter - periplasmic binding protein | 0.026 | 1.09E-04 |
| b2239 | *glpQ* | glycerophosphoryl diester phosphodiesterase, periplasmic | 0.024 | 3.60E-07 |
| b2241 | *glpA* | glycerol-3-phosphate dehydrogenase (anaerobic), large subunit | 0.023 | 1.81E-07 |
| b4035 | *malK* | maltose ABC transporter - ATP binding subunit | 0.022 | 4.09E-07 |
| b3225 | *nanA* | N-acetylneuraminate lyase | 0.020 | 1.36E-06 |
| b2702 | *srlA* | glucitol/sorbitol-specific enzyme IIC component of PTS | 0.017 | 1.08E-03 |
| b3223 | *nanE* | predicted N-acetylmannosamine-6-phosphate epimerase | 0.014 | 5.95E-03 |
| b2240 | *glpT* | GlpT glycerol-3-P MFS transporter | 0.014 | 1.43E-06 |
| b3709 | *tnaB* | TnaB tryptophan ArAAP transporter | 0.012 | 5.74E-07 |
| b3117 | *tdcB* | catabolic threonine dehydratase | 0.008 | 5.33E-07 |
| b3708 | *tnaA* | L-cysteine desulfhydrase / tryptophanase | 0.004 | 1.66E-07 |

^a^From the EcoCyc database (http://ecocyc.org)

^b^Fold-change in gene expression between iE2 and BW25113 (average of triplicate experiments)
