# Supplementary material for: Improving Ethanol Tolerance of Escherichia coli by Rewiring Its Global Regulator cAMP Receptor Protein (CRP)
Source: PLoS One. 2013 Feb 28;8(2):e57628. doi: 10.1371/journal.pone.0057628 (PMC3585226; doi:10.1371/journal.pone.0057628)
Supplement: Table S3 — Genes with >2-fold change in their expression level in iE2 as compared to BW25113 in the prence of ethanol stress, using a p-value threshold less than 0.05. (DOCX) [file pone.0057628.s004.docx]

**TABLE S3.** Genes with >2-fold change in their expression level in iE2 as compared to BW25113 in the prence of ethanol stress, using a *p*-value threshold less than 0.05.

| **b-number** | **Gene** | **Function^a^** | **Fold-change^b^** | ***p*-value** |
| --- | --- | --- | --- | --- |
| b0759 | *galE* | UDP-glucose 4-epimerase | 22.301 | 1.38E-03 |
| b3114 | *tdcE* | 2-ketobutyrate formate-lyase/pyruvate formate-lyase 4, inactive | 11.194 | 1.89E-06 |
| b4068 | *yjcH* | conserved inner membrane protein | 8.782 | 1.29E-03 |
| b3452 | *ugpA* | glycerol-3-phosphate / glycerol-2-phosphate ABC transporter - putative membrane subunit | 8.772 | 1.65E-04 |
| b3581 | *sgbH* | 3-keto-L-gulonate 6-phosphate decarboxylase | 8.016 | 1.67E-02 |
| b3223 | *nanE* | predicted N-acetylmannosamine-6-phosphate epimerase | 7.062 | 6.27E-04 |
| b3750 | *rbsC* | ribose ABC transporter - membrane subunit | 6.573 | 1.35E-03 |
| b0036 | *caiD* | crotonobetainyl-CoA hydratase | 6.372 | 2.71E-05 |
| b3513 | *mdtE* | MdtEF-TolC multidrug efflux transport system - membrane fusion protein | 5.532 | 1.67E-04 |
| b3748 | *rbsD* | ribose pyranase | 4.834 | 3.77E-04 |
| b3392 | *hofP* | protein involved in utilization of DNA as a carbon source | 4.648 | 8.20E-05 |
| b4288 | *fecD* | ferric dicitrate ABC transporter - membrane subunit | 4.400 | 1.05E-03 |
| b3709 | *tnaB* | TnaB tryptophan ArAAP transporter | 4.369 | 9.99E-03 |
| b3907 | *rhaT* | rhamnose RhaT transporter | 4.340 | 1.53E-04 |
| b2489 | *hyfI* | hydrogenase 4, small subunit | 4.251 | 1.80E-02 |
| b4289 | *fecC* | ferric dicitrate ABC transporter - membrane subunit | 3.930 | 7.26E-03 |
| b3393 | *hofO* | protein involved in utilization of DNA as a carbon source | 3.772 | 1.09E-03 |
| b3366 | *nirD* | nitrite reductase, small subunit | 3.762 | 4.96E-02 |
| b1900 | *araG* | arabinose ABC transporter - ATP binding subunit | 3.693 | 4.32E-03 |
| b0431 | *cyoB* | cytochrome bo terminal oxidase subunit I | 3.661 | 1.85E-03 |
| b3224 | *nanT* | NanT sialic acid MFS transporter | 3.637 | 2.73E-03 |
| b1394 | *paaG* | predicted ring 1,2-epoxyphenylacetyl-CoA isomerase (oxepin-CoA forming) | 3.622 | 4.39E-02 |
| b0035 | *caiE* | predicted acyl transferase | 3.578 | 3.08E-02 |
| b3588 | *aldB* | acetaldehyde dehydrogenase | 3.454 | 6.76E-04 |
| b3365 | *nirB* | dimer of large subunit of nitrite reductase | 3.370 | 1.57E-05 |
| b0553 | *nmpC* | outer membrane porin protein; locus of qsr prophage | 3.344 | 1.84E-04 |
| b3116 | *tdcC* | TdcC threonine STP transporter | 3.338 | 2.82E-04 |
| b2148 | *mglC* | galactose ABC transporter - membrane subunit | 3.316 | 2.38E-03 |
| b3580 | *lyxK* | L-xylulose kinase | 3.237 | 4.64E-02 |
| b3221 | *yhcH* | conserved protein | 3.175 | 2.76E-04 |
| b1531 | *marA* | MarA DNA-binding transcriptional dual regulator | 3.072 | 8.82E-08 |
| b4287 | *fecE* | ferric dicitrate ABC transporter - ATP binding subunit | 2.947 | 6.08E-04 |
| b1389 | *paaB* | ring 1,2-phenylacetyl-CoA epoxidase subunit | 2.930 | 1.76E-02 |
| b3566 | *xylF* | xylose ABC transporter - periplasmic binding protein | 2.884 | 7.67E-03 |
| b3115 | *tdcD* | propionate kinase | 2.861 | 1.25E-04 |
| b1615 | *uidC* | membrane-associated protein | 2.857 | 1.92E-02 |
| b2492 | *focB* | FocB formate FNT transporter | 2.827 | 2.05E-02 |
| b2535 | *csiE* | stationary phase inducible protein | 2.797 | 2.20E-05 |
| b0034 | *caiF* | CaiF transcriptional activator | 2.784 | 7.45E-03 |
| b3394 | *hofN* | protein involved in utilization of DNA as a carbon source | 2.743 | 2.00E-04 |
| b3437 | *gntK* | D-gluconate kinase, thermostable | 2.737 | 3.47E-05 |
| b0723 | *sdhA* | succinate dehydrogenase flavoprotein | 2.728 | 1.41E-02 |
| b3721 | *bglB* | 6-phospho-β-glucosidase B | 2.710 | 6.48E-03 |
| b2147 | *preA* | NADH-dependent dihydropyrimidine dehydrogenase subunit | 2.663 | 3.33E-02 |
| b0598 | *cstA* | peptide transporter induced by carbon starvation | 2.646 | 3.71E-02 |
| b4268 | *idnK* | D-gluconate kinase, thermosensitive | 2.642 | 3.17E-03 |
| b4197 | *ulaE* | L-xylulose 5-phosphate 3-epimerase | 2.576 | 1.34E-02 |
| b0429 | *cyoD* | cytochrome bo terminal oxidase subunit IV | 2.561 | 7.16E-04 |
| b3117 | *tdcB* | catabolic threonine dehydratase | 2.544 | 2.48E-02 |
| b4265 | *idnT* | L-idonate / 5-ketogluconate / gluconate transporter | 2.537 | 2.24E-05 |
| b2150 | *mglB* | galactose ABC transporter - periplasmic binding protein | 2.535 | 3.96E-02 |
| b1112 | *bhsA* | protein involved in stress resistance and biofilm formation | 2.528 | 3.19E-07 |
| b1517 | *lsrF* | predicted class I aldolase | 2.507 | 2.66E-02 |
| b3904 | *rhaB* | L-rhamnulose kinase | 2.411 | 4.78E-02 |
| b2487 | *hyfG* | hydrogenase 4, large subunit | 2.403 | 1.61E-04 |
| b2803 | *fucK* | L-fuculokinase | 2.399 | 6.65E-04 |
| b0583 | *entD* | phosphopantetheinyl transferase | 2.387 | 5.74E-03 |
| b4471 | *tdcG* | L-serine deaminase III | 2.381 | 5.72E-03 |
| b3222 | *nanK* | N-acetylmannosamine kinase | 2.376 | 3.22E-04 |
| b2243 | *glpC* | glycerol-3-phosphate dehydrogenase (anaerobic), small subunit | 2.359 | 3.99E-03 |
| b0346 | *mhpR* | MhpR transcriptional activator | 2.314 | 4.98E-03 |
| b1518 | *lsrG* | Autoinducer 2-degrading protein | 2.272 | 7.91E-03 |
| b4193 | *ulaA* | L-ascorbate-specific enzyme IIC component of PTS | 2.248 | 1.59E-03 |
| b1040 | *csgD* | CsgD DNA-binding transcriptional dual regulator | 2.234 | 4.00E-02 |
| b4376 | *osmY* | periplasmic protein | 2.179 | 9.92E-03 |
| b3751 | *rbsB* | ribose ABC transporter - putative periplasmic binding protein | 2.150 | 1.27E-02 |
| b3578 | *yiaN* | L-dehydroascorbate transporter | 2.139 | 1.86E-03 |
| b1519 | *tam* | trans-aconitate methyltransferase | 2.118 | 1.24E-03 |
| b2469 | *narQ* | NarQ sensory histidine kinase | 2.090 | 6.82E-04 |
| b2393 | *nupC* | NupC nucleoside NUP transporter | 2.083 | 1.09E-03 |
| b3582 | *sgbU* | predicted L-xylulose 5-phosphate 3-epimerase | 2.073 | 2.90E-02 |
| b3260 | *dusB* | tRNA dihydrouridine synthase | 0.494 | 1.02E-06 |
| b1819 | *manZ* | mannose PTS permease - ManZ subunit | 0.477 | 9.41E-06 |
| b2092 | *gatC* | galactitol-specific enzyme IIC component of PTS | 0.459 | 9.40E-03 |
| b4266 | *idnO* | 5-keto-D-gluconate 5-reductase | 0.447 | 4.88E-03 |
| b2913 | *serA* | α-ketoglutarate reductase / D-3-phosphoglycerate dehydrogenase | 0.444 | 2.69E-05 |
| b0350 | *mhpD* | 2-oxopent-4-enoate hydratase | 0.426 | 1.54E-04 |
| b4460 | *araH* | arabinose ABC transporter - membrane subunit | 0.415 | 6.37E-03 |
| b0428 | *cyoE* | heme O synthase | 0.407 | 1.36E-03 |
| b3165 | *rpsO* | 30S ribosomal subunit protein S15 | 0.391 | 4.81E-07 |
| b4118 | *melR* | MelR DNA-binding transcriptional dual regulator | 0.389 | 2.44E-05 |
| b2663 | *gabP* | GabP APC transporter | 0.373 | 4.16E-02 |
| b3367 | *nirC* | NirC nitrite FNT transporter | 0.373 | 5.00E-03 |
| b0675 | *nagD* | ribonucleotide monophosphatase | 0.355 | 1.13E-04 |
| b3213 | *gltD* | glutamate synthase, small subunit | 0.328 | 3.12E-05 |
| b4194 | *ulaB* | L-ascorbate-specific enzyme IIB component of PTS | 0.281 | 6.21E-04 |
| b0593 | *entC* | isochorismate synthase 1 | 0.258 | 5.81E-04 |
| b1101 | *ptsG* | fused glucose-specific PTS enzymes: IIB component/IIC component | 0.233 | 1.11E-05 |
| b2703 | *srlE* | glucitol/sorbitol-specific enzyme IIB component of PTS | 0.062 | 1.51E-04 |
| b2702 | *srlA* | glucitol/sorbitol-specific enzyme IIC component of PTS | 0.028 | 2.39E-08 |
| b2705 | *srlD* | sorbitol-6-phosphate dehydrogenase | 0.026 | 1.64E-05 |
| b2704 | *srlB* | glucitol/sorbitol-specific enzyme IIA component of PTS | 0.025 | 4.46E-05 |

^a^From the EcoCyc database (http://ecocyc.org)

^b^Fold-change in gene expression between iE2 and BW25113 (average of triplicate experiments)
