# Supplementary material for: Improving Ethanol Tolerance of Escherichia coli by Rewiring Its Global Regulator cAMP Receptor Protein (CRP)
Source: PLoS One. 2013 Feb 28;8(2):e57628. doi: 10.1371/journal.pone.0057628 (PMC3585226; doi:10.1371/journal.pone.0057628)
Supplement: Table S4 — Genes with >2-fold change in their expression level in E2 as compared to the control in the absence of ethanol stress, using a p-value threshold less than 0.05. (DOCX) [file pone.0057628.s005.docx]

**TABLE S4.** Genes with >2-fold change in their expression level in E2 as compared to the control in the absence of ethanol stress, using a *p*-value threshold less than 0.05.

| **b-number** | **Gene** | **Function^a^** | **Fold-change^b^** | ***p*-value** |
| --- | --- | --- | --- | --- |
| b1493 | *gadB* | glutamate decarboxylase B | 7.246 | 2.34E-08 |
| b2393 | *nupC* | NupC nucleoside NUP transporter | 6.749 | 4.27E-07 |
| b1492 | *gadC* | GadC GABA APC transporter | 6.587 | 4.07E-08 |
| b2508 | *guaB* | IMP dehydrogenase | 4.841 | 1.26E-06 |
| b2507 | *guaA* | GMP synthetase | 4.564 | 2.82E-06 |
| b0431 | *cyoB* | cytochrome bo terminal oxidase subunit I | 3.623 | 2.62E-07 |
| b3512 | *gadE* | GadE DNA-binding transcriptional activator | 3.616 | 1.07E-04 |
| b1101 | *ptsG* | fused glucose-specific PTS enzymes: IIB component/IIC component | 3.291 | 1.68E-06 |
| b0428 | *cyoE* | heme O synthase | 3.117 | 2.25E-06 |
| b0037 | *caiC* | carnitine-CoA ligase | 2.920 | 2.91E-02 |
| b0597 | *entH* | proofreading thioesterase in enterobactin biosynthesis | 2.897 | 4.61E-06 |
| b3513 | *mdtE* | MdtEF-TolC multidrug efflux transport system - membrane fusion protein | 2.827 | 1.53E-04 |
| b1272 | *sohB* | predicted inner membrane peptidase | 2.822 | 1.48E-07 |
| b0430 | *cyoC* | cytochrome bo terminal oxidase subunit III | 2.764 | 9.71E-08 |
| b3517 | *gadA* | glutamate decarboxylase A | 2.690 | 8.59E-07 |
| b2235 | *nrdB* | ribonucleoside diphosphate reductase 1, β subunit dimer | 2.578 | 4.80E-05 |
| b0429 | *cyoD* | cytochrome bo terminal oxidase subunit IV | 2.537 | 2.82E-06 |
| b4291 | *fecA* | outer membrane receptor; citrate-dependent iron transport, outer membrane receptor | 2.487 | 1.05E-03 |
| b1038 | *csgF* | curli assembly component | 2.473 | 2.49E-02 |
| b2171 | *yeiP* | predicted dehydrogenase, NAD-dependent | 2.449 | 6.40E-06 |
| b0343 | *lacY* | LacY lactose MFS transporter | 2.441 | 3.52E-02 |
| b1395 | *paaH* | 3-hydroxyadipyl-CoA dehydrogenase (NAD+) | 2.419 | 6.77E-04 |
| b2942 | *metK* | methionine adenosyltransferase | 2.244 | 5.04E-05 |
| b3806 | *cyaA* | adenylate cyclase | 2.212 | 1.89E-06 |
| b3261 | *fis* | Fis DNA-binding transcriptional dual regulator | 2.162 | 1.53E-05 |
| b0907 | *serC* | phosphohydroxythreonine aminotransferase / 3-phosphoserine aminotransferase | 2.129 | 2.06E-07 |
| b2488 | *hyfH* | hydrogenase 4, component H | 2.115 | 3.73E-05 |
| b3260 | *dusB* | tRNA dihydrouridine synthase | 2.093 | 1.44E-04 |
| b3516 | *gadX* | GadX DNA-binding transcriptional dual regulator | 2.078 | 9.67E-05 |
| b0595 | *entB* | apo-EntB multimer | 2.059 | 8.35E-04 |
| b2344 | *fadL* | long-chain fatty acid outer membrane transporter; sensitivity to phage T2 | 2.048 | 9.40E-09 |
| b0411 | *tsx* | nucleoside channel; receptor of phage T6 and colicin K | 2.047 | 1.90E-08 |
| b0125 | *hpt* | hypoxanthine phosphoribosyltransferase | 2.017 | 7.39E-10 |
| b0106 | *hofC* | protein transport protein HofC | 2.003 | 3.66E-03 |
| b2492 | *focB* | FocB formate FNT transporter | 0.499 | 1.84E-04 |
| b2705 | *srlD* | sorbitol-6-phosphate dehydrogenase | 0.496 | 7.17E-04 |
| b0679 | *nagE* | N-acetylglucosamine PTS permease | 0.489 | 6.39E-07 |
| b3961 | *oxyR* | OxyR DNA-binding transcriptional dual regulator | 0.488 | 7.90E-04 |
| b0036 | *caiD* | crotonobetainyl-CoA hydratase | 0.483 | 1.01E-02 |
| b1531 | *marA* | MarA DNA-binding transcriptional dual regulator | 0.481 | 5.91E-09 |
| b3751 | *rbsB* | ribose ABC transporter - putative periplasmic binding protein | 0.477 | 6.64E-07 |
| b3905 | *rhaS* | RhaS transcriptional activator | 0.475 | 2.97E-02 |
| b2804 | *fucU* | L-fucose mutarotase | 0.474 | 1.98E-03 |
| b2957 | *ansB* | asparaginase II | 0.473 | 1.00E-05 |
| b3453 | *ugpB* | glycerol-3-phosphate / glycerol-2-phosphate ABC transporter - putative periplasmic binding protein | 0.468 | 4.79E-02 |
| b3414 | *nfuA* | iron-sulfur cluster scaffold protein | 0.467 | 3.58E-08 |
| b3430 | *glgC* | glucose-1-phosphate adenylyltransferase | 0.466 | 1.18E-07 |
| b3425 | *glpE* | thiosulfate sulfurtransferase | 0.465 | 1.85E-04 |
| b2093 | *gatB* | galactitol-specific enzyme IIB component of PTS | 0.457 | 9.78E-06 |
| b4194 | *ulaB* | L-ascorbate-specific enzyme IIB component of PTS | 0.456 | 9.52E-05 |
| b1594 | *dgsA* | DgsA DNA-binding transcriptional repressor | 0.454 | 4.27E-06 |
| b0721 | *sdhC* | succinate dehydrogenase membrane protein | 0.449 | 1.22E-08 |
| b2659 | *csiD* | predicted protein | 0.444 | 7.58E-03 |
| b4197 | *ulaE* | L-xylulose 5-phosphate 3-epimerase | 0.442 | 4.01E-02 |
| b4213 | *cpdB* | 2',3'-cyclic nucleotide 2'-phosphodiesterase / 3'-nucleotidase | 0.435 | 4.99E-06 |
| b2801 | *fucP* | FucP fucose MFS transporter | 0.433 | 1.97E-02 |
| b3582 | *sgbU* | predicted L-xylulose 5-phosphate 3-epimerase | 0.429 | 4.09E-02 |
| b1620 | *malI* | MalI DNA-binding transcriptional repressor | 0.428 | 6.69E-09 |
| b2092 | *gatC* | galactitol-specific enzyme IIC component of PTS | 0.424 | 3.08E-11 |
| b3750 | *rbsC* | ribose ABC transporter - membrane subunit | 0.424 | 6.98E-03 |
| b3868 | *glnG* | NtrC transcriptional dual regulator | 0.422 | 2.06E-03 |
| b4003 | *zraS* | ZraS sensory histidine kinase | 0.418 | 5.60E-03 |
| b0764 | *modB* | molybdate ABC transporter - membrane subunit | 0.414 | 2.12E-06 |
| b1112 | *bhsA* | protein involved in stress resistance and biofilm formation | 0.413 | 2.04E-04 |
| b3461 | *rpoH* | RNA polymerase, sigma 32 (sigma H) factor | 0.404 | 5.73E-06 |
| b3236 | *mdh* | malate dehydrogenase | 0.396 | 3.18E-06 |
| b4239 | *treC* | trehalose-6-phosphate hydrolase | 0.395 | 5.89E-08 |
| b4004 | *zraR* | ZraR transcriptional activator | 0.395 | 7.73E-03 |
| b3426 | *glpD* | glycerol 3-phosphate dehydrogenase, aerobic | 0.392 | 5.37E-04 |
| b3077 | *ebgC* | evolved β-D-galactosidase, β subunit | 0.390 | 1.16E-02 |
| b2803 | *fucK* | L-fuculokinase | 0.383 | 1.54E-05 |
| b2704 | *srlB* | glucitol/sorbitol-specific enzyme IIA component of PTS | 0.381 | 1.76E-04 |
| b2805 | *fucR* | FucR transcriptional activator | 0.380 | 1.32E-07 |
| b2660 | *lhgO* | L-2-hydroxyglutarate oxidase | 0.380 | 1.92E-02 |
| b3091 | *uxaA* | D-altronate dehydratase | 0.379 | 1.09E-07 |
| b3748 | *rbsD* | ribose pyranase | 0.378 | 1.44E-06 |
| b3588 | *aldB* | acetaldehyde dehydrogenase | 0.376 | 3.92E-07 |
| b0722 | *sdhD* | succinate dehydrogenase membrane protein | 0.371 | 1.11E-11 |
| b1817 | *manX* | mannose PTS permease - ManX subunit | 0.369 | 2.26E-11 |
| b3241 | *aaeA* | AaeAB Hydroxylated, Aromatic Carboxylic Acid Efflux Transport System Protein A | 0.365 | 2.31E-02 |
| b3134 | *agaW* | PTS system N-acetylgalactosameine-specific IIC component 2 | 0.360 | 1.22E-02 |
| b3093 | *exuT* | ExuT hexuronate MFS transporter | 0.359 | 2.11E-04 |
| b4265 | *idnT* | L-idonate / 5-ketogluconate / gluconate transporter | 0.352 | 1.47E-03 |
| b2365 | *dsdX* | DsdX Gnt tranporter | 0.348 | 8.01E-11 |
| b3092 | *uxaC* | D-glucuronate isomerase / D-galacturonate isomerase | 0.347 | 4.43E-11 |
| b3415 | *gntT* | GntT Gluconate Gnt transporter | 0.344 | 1.18E-03 |
| b1615 | *uidC* | membrane-associated protein | 0.339 | 1.84E-03 |
| b3576 | *yiaL* | conserved protein | 0.339 | 8.29E-03 |
| b1002 | *agp* | 3-phytase / glucose-1-phosphatase | 0.327 | 2.36E-08 |
| b2800 | *fucA* | L-fuculose-phosphate aldolase | 0.325 | 2.19E-03 |
| b4193 | *ulaA* | L-ascorbate-specific enzyme IIC component of PTS | 0.323 | 1.49E-02 |
| b0061 | *araD* | L-ribulose 5-phosphate 4-epimerase | 0.322 | 1.15E-02 |
| b2715 | *ascF* | β-glucoside PTS permease | 0.319 | 3.76E-02 |
| b4119 | *melA* | α-galactosidase | 0.315 | 3.87E-03 |
| b0720 | *gltA* | citrate synthase | 0.310 | 8.40E-07 |
| b4037 | *malM* | maltose regulon periplasmic protein | 0.308 | 3.06E-06 |
| b4118 | *melR* | MelR DNA-binding transcriptional dual regulator | 0.308 | 8.53E-04 |
| b4033 | *malF* | maltose ABC transporter - membrane subunit | 0.306 | 2.02E-08 |
| b2091 | *gatD* | galactitol-1-phosphate dehydrogenase | 0.302 | 2.78E-12 |
| b3670 | *ilvN* | acetolactate synthase I, small subunit | 0.301 | 1.26E-07 |
| b4034 | *malE* | maltose ABC transporter - periplasmic binding protein | 0.284 | 2.48E-09 |
| b3113 | *tdcF* | predicted L-PSP (mRNA) endoribonuclease | 0.283 | 5.40E-08 |
| b4032 | *malG* | maltose ABC transporter - membrane subunit | 0.283 | 1.25E-06 |
| b3583 | *sgbE* | L-ribulose-5-phosphate 4-epimerase | 0.282 | 1.47E-02 |
| b3081 | *fadH* | 2,4-dienoyl-CoA reductase | 0.277 | 1.99E-03 |
| b2802 | *fucI* | L-fucose isomerase | 0.270 | 5.28E-06 |
| b4196 | *ulaD* | 3-keto-L-gulonate 6-phosphate decarboxylase | 0.269 | 1.95E-02 |
| b4321 | *gntP* | GntP Gluconate Gnt transporter | 0.267 | 2.05E-04 |
| b4266 | *idnO* | 5-keto-D-gluconate 5-reductase | 0.267 | 1.82E-02 |
| b4035 | *malK* | maltose ABC transporter - ATP binding subunit | 0.266 | 1.78E-04 |
| b4036 | *lamB* | phage lambda receptor protein; maltose high-affinity receptor | 0.264 | 3.76E-05 |
| b0064 | *araC* | AraC DNA-binding transcriptional dual regulator | 0.262 | 3.49E-10 |
| b3133 | *agaV* | PTS system, cytoplasmic, N-acetylgalactosamine-specific IIB component 2 (EIIB-AGA) | 0.258 | 4.05E-04 |
| b3565 | *xylA* | xylose isomerase | 0.249 | 2.09E-04 |
| b3114 | *tdcE* | 2-ketobutyrate formate-lyase/pyruvate formate-lyase 4, inactive | 0.249 | 5.44E-07 |
| b4240 | *treB* | fused trehalose(maltose)-specific PTS enzyme: IIB component/IIC component | 0.248 | 5.87E-10 |
| b1205 | *ychH* | stress-induced protein | 0.246 | 3.67E-09 |
| b2841 | *araE* | AraE arabinose MFS transporter | 0.245 | 3.74E-08 |
| b1190 | *dadX* | alanine racemase 2, PLP-binding | 0.244 | 3.37E-06 |
| b2799 | *fucO* | L-1,2-propanediol oxidoreductase | 0.244 | 8.12E-05 |
| b3926 | *glpK* | glycerol kinase | 0.227 | 1.15E-06 |
| b2239 | *glpQ* | glycerophosphoryl diester phosphodiesterase, periplasmic | 0.227 | 3.85E-08 |
| b4268 | *idnK* | D-gluconate kinase, thermosensitive | 0.221 | 7.33E-04 |
| b3221 | *yhcH* | conserved protein | 0.220 | 2.50E-08 |
| b2240 | *glpT* | GlpT glycerol-3-P MFS transporter | 0.220 | 2.00E-09 |
| b0113 | *pdhR* | PdhR DNA-binding transcriptional dual regulator | 0.217 | 8.59E-13 |
| b3566 | *xylF* | xylose ABC transporter - periplasmic binding protein | 0.217 | 1.34E-04 |
| b3671 | *ilvB* | acetolactate synthase I, large subunit | 0.212 | 3.22E-07 |
| b4460 | *araH* | arabinose ABC transporter - membrane subunit | 0.211 | 1.16E-11 |
| b3076 | *ebgA* | evolved β-D-galactosidase, α subunit | 0.210 | 2.16E-04 |
| b2614 | *grpE* | phage lambda replication; host DNA synthesis; heat shock protein; protein repair | 0.201 | 5.67E-06 |
| b2148 | *mglC* | galactose ABC transporter - membrane subunit | 0.198 | 3.68E-07 |
| b3575 | *yiaK* | 2,3-diketo-L-gulonate reductase | 0.197 | 5.06E-03 |
| b4310 | *nanM* | N-acetylneuraminate mutarotase | 0.193 | 2.17E-06 |
| b3581 | *sgbH* | 3-keto-L-gulonate 6-phosphate decarboxylase | 0.186 | 1.24E-02 |
| b4322 | *uxuA* | D-mannonate dehydratase | 0.186 | 9.73E-12 |
| b3528 | *dctA* | DctA dicarboxylate DAACS transporter | 0.179 | 4.19E-07 |
| b4067 | *actP* | acetate / glycolate transporter | 0.177 | 1.50E-04 |
| b4267 | *idnD* | L-idonate 5-dehydrogenase | 0.167 | 1.88E-02 |
| b1415 | *aldA* | aldehyde dehydrogenase A, NAD-linked | 0.159 | 1.83E-08 |
| b4311 | *nanC* | N-acetylneuraminic acid outer membrane channel | 0.153 | 2.37E-07 |
| b1189 | *dadA* | D-amino acid dehydrogenase | 0.146 | 3.49E-09 |
| b0346 | *mhpR* | MhpR transcriptional activator | 0.139 | 9.43E-06 |
| b1901 | *araF* | arabinose ABC transporter - periplasmic binding protein | 0.136 | 1.11E-06 |
| b0723 | *sdhA* | succinate dehydrogenase flavoprotein | 0.135 | 3.54E-09 |
| b4068 | *yjcH* | conserved inner membrane protein | 0.133 | 8.15E-05 |
| b3225 | *nanA* | N-acetylneuraminate lyase | 0.132 | 4.87E-05 |
| b1900 | *araG* | arabinose ABC transporter - ATP binding subunit | 0.131 | 6.91E-06 |
| b2151 | *galS* | GalS DNA-binding transcriptional dual regulator | 0.124 | 1.09E-04 |
| b2146 | *preT* | NADH-dependent dihydropyrimidine dehydrogenase subunit | 0.123 | 2.36E-09 |
| b3118 | *tdcA* | TdcA DNA-binding transcriptional activator | 0.122 | 5.38E-07 |
| b3927 | *glpF* | GlpF glycerol MIP channel | 0.119 | 3.15E-05 |
| b3222 | *nanK* | N-acetylmannosamine kinase | 0.109 | 1.56E-06 |
| b2147 | *preA* | NADH-dependent dihydropyrimidine dehydrogenase subunit | 0.099 | 2.51E-05 |
| b0596 | *entA* | 2,3-dihydro-2,3-dihydroxybenzoate dehydrogenase | 0.096 | 6.00E-03 |
| b2149 | *mglA* | galactose ABC transporter - ATP binding subunit | 0.090 | 3.49E-08 |
| b4323 | *uxuB* | D-mannonate oxidoreductase | 0.082 | 6.90E-06 |
| b3224 | *nanT* | NanT sialic acid MFS transporter | 0.078 | 4.66E-08 |
| b2980 | *glcC* | GlcC transcriptional dual regulator | 0.075 | 6.88E-04 |
| b0598 | *cstA* | peptide transporter induced by carbon starvation | 0.063 | 8.00E-05 |
| b3116 | *tdcC* | TdcC threonine STP transporter | 0.051 | 5.47E-06 |
| b3117 | *tdcB* | catabolic threonine dehydratase | 0.040 | 4.58E-04 |
| b3709 | *tnaB* | TnaB tryptophan ArAAP transporter | 0.039 | 4.95E-07 |
| b3223 | *nanE* | predicted N-acetylmannosamine-6-phosphate epimerase | 0.035 | 1.33E-02 |
| b2150 | *mglB* | galactose ABC transporter - periplasmic binding protein | 0.030 | 1.22E-06 |
| b3115 | *tdcD* | propionate kinase | 0.026 | 1.44E-07 |
| b3708 | *tnaA* | L-cysteine desulfhydrase / tryptophanase | 0.019 | 1.78E-06 |
| b4069 | *acs* | acetyl-CoA synthetase (AMP-forming) | 0.014 | 4.57E-07 |
| b0759 | *galE* | UDP-glucose 4-epimerase | 0.001 | 1.14E-04 |

^a^From the EcoCyc database (http://ecocyc.org)

^b^Fold change in gene expression between E2 and the control (average of duplicate experiments)
