# Supplementary material for: Improving Ethanol Tolerance of Escherichia coli by Rewiring Its Global Regulator cAMP Receptor Protein (CRP)
Source: PLoS One. 2013 Feb 28;8(2):e57628. doi: 10.1371/journal.pone.0057628 (PMC3585226; doi:10.1371/journal.pone.0057628)
Supplement: Table S5 — Genes with >2-fold change in their expression level in E2 as compared to the control in the presence of ethanol stress, using a p-value threshold less than 0.05. (DOCX) [file pone.0057628.s006.docx]

**TABLE S5.** Genes with >2-fold change in their expression level in E2 as compared to the control in the presence of ethanol stress, using a *p*-value threshold less than 0.05.

| **b-number** | **Gene** | **Function^a^** | **Fold-change^b^** | ***p*-value** |
| --- | --- | --- | --- | --- |
| b0759 | *galE* | UDP-glucose 4-epimerase | 265.076 | 2.66E-02 |
| b0041 | *fixA* | probable flavoprotein subunit required for anaerobic carnitine metabolism | 27.385 | 3.35E-03 |
| b0040 | *caiT* | L-carnitine/γ-butyrobetaine antiport | 8.127 | 2.36E-02 |
| b0596 | *entA* | 2,3-dihydro-2,3-dihydroxybenzoate dehydrogenase | 7.540 | 7.89E-05 |
| b0334 | *prpD* | 2-methylcitrate dehydratase | 6.639 | 1.79E-02 |
| b1112 | *bhsA* | protein involved in stress resistance and biofilm formation | 4.061 | 2.38E-05 |
| b0594 | *entE* | 2,3-dihydroxybenzoate-holo-EntB ligase [multifunctional] | 3.766 | 1.65E-05 |
| b1779 | *gapA* | glyceraldehyde 3-phosphate dehydrogenase-A complex | 3.355 | 9.08E-13 |
| b0764 | *modB* | molybdate ABC transporter - membrane subunit | 3.217 | 1.35E-03 |
| b0429 | *cyoD* | cytochrome bo terminal oxidase subunit IV | 3.050 | 2.61E-06 |
| b0428 | *cyoE* | heme O synthase | 2.780 | 1.91E-02 |
| b0432 | *cyoA* | cytochrome bo terminal oxidase subunit II | 2.757 | 4.46E-05 |
| b1531 | *marA* | MarA DNA-binding transcriptional dual regulator | 2.700 | 8.06E-04 |
| b0431 | *cyoB* | cytochrome bo terminal oxidase subunit I | 2.641 | 4.38E-02 |
| b0124 | *gcd* | glucose dehydrogenase | 2.543 | 3.24E-03 |
| b0125 | *hpt* | hypoxanthine phosphoribosyltransferase | 2.537 | 9.19E-05 |
| b0038 | *caiB* | γ-butyrobetainyl-CoA:carnitine CoA transferase | 2.430 | 2.48E-02 |
| b0118 | *acnB* | bifunctional aconitate hydratase 2 and 2-methylisocitrate dehydratase | 2.393 | 2.54E-06 |
| b4015 | *aceA* | isocitrate lyase | 2.376 | 3.33E-04 |
| b0344 | *lacZ* | β-galactosidase | 2.337 | 1.92E-03 |
| b2171 | *yeiP* | predicted dehydrogenase, NAD-dependent | 2.310 | 4.94E-04 |
| b0116 | *lpd* | lipoamide dehydrogenase | 2.213 | 7.25E-04 |
| b2925 | *fbaA* | fructose bisphosphate aldolase class II | 2.180 | 5.79E-04 |
| b3868 | *sodA* | superoxide dismutase (Mn) | 2.144 | 1.51E-03 |
| b0595 | *entB* | apo-EntB multimer | 2.090 | 1.64E-03 |
| b0583 | *entD* | phosphopantetheinyl transferase | 2.089 | 6.92E-03 |
| b0430 | *cyoC* | cytochrome bo terminal oxidase subunit III | 2.039 | 5.79E-07 |
| b4016 | *aceK* | isocitrate dehydrogenase phosphatase / isocitrate dehydrogenase kinase | 2.007 | 9.27E-04 |
| b2151 | *galS* | GalS DNA-binding transcriptional dual regulator | 0.499 | 3.08E-02 |
| b3425 | *glpE* | thiosulfate sulfurtransferase | 0.491 | 1.52E-05 |
| b4034 | *malE* | maltose ABC transporter - periplasmic binding protein | 0.491 | 2.20E-02 |
| b1512 | *lsrR* | LsrR DNA-binding transcriptional repressor | 0.491 | 4.03E-03 |
| b4382 | *deoA* | thymidine phosphorylase | 0.489 | 7.51E-04 |
| b2803 | *fucK* | L-fuculokinase | 0.486 | 3.51E-02 |
| b0106 | *hofC* | protein transport protein HofC | 0.485 | 1.30E-03 |
| b1521 | *uxaB* | altronate oxidoreductase | 0.482 | 1.28E-03 |
| b4268 | *idnK* | D-gluconate kinase, thermosensitive | 0.479 | 3.38E-03 |
| b3749 | *rbsA* | ribose ABC transporter - putative ATP binding subunit | 0.478 | 1.22E-03 |
| b3751 | *rbsB* | ribose ABC transporter - putative periplasmic binding protein | 0.476 | 5.78E-03 |
| b3367 | *nirC* | NirC nitrite FNT transporter | 0.476 | 2.61E-02 |
| b2964 | *nupG* | NupG nucleoside MFS transporter | 0.476 | 1.07E-02 |
| b3423 | *glpR* | GlpR DNA-binding transcriptional repressor | 0.475 | 7.46E-05 |
| b1616 | *uidB* | UidB glucuronides GPH transporter | 0.474 | 3.61E-03 |
| b3564 | *xylB* | xylulokinase | 0.474 | 1.53E-02 |
| b4321 | *gntP* | GntP Gluconate Gnt transporter | 0.471 | 3.31E-04 |
| b1594 | *dgsA* | DgsA DNA-binding transcriptional repressor | 0.470 | 1.38E-04 |
| b4123 | *dcuB* | DcuB dicarboxylate Dcu transporter | 0.463 | 4.73E-02 |
| b2715 | *ascF* | β-glucoside PTS permease | 0.460 | 2.50E-03 |
| b2957 | *ansB* | asparaginase II | 0.459 | 1.88E-02 |
| b4239 | *treC* | trehalose-6-phosphate hydrolase | 0.455 | 1.33E-02 |
| b3424 | *glpG* | intramembrane serine protease GlpG | 0.454 | 3.26E-04 |
| b4194 | *ulaB* | L-ascorbate-specific enzyme IIB component of PTS | 0.453 | 3.20E-03 |
| b4139 | *aspA* | aspartate ammonia-lyase | 0.450 | 4.53E-04 |
| b4289 | *fecC* | ferric dicitrate ABC transporter - membrane subunit | 0.450 | 2.51E-02 |
| b0346 | *mhpR* | MhpR transcriptional activator | 0.448 | 3.89E-04 |
| b0908 | *aroA* | 3-phosphoshikimate-1-carboxyvinyltransferase | 0.441 | 5.35E-03 |
| b2149 | *mglA* | galactose ABC transporter - ATP binding subunit | 0.427 | 9.55E-03 |
| b1389 | *paaB* | ring 1,2-phenylacetyl-CoA epoxidase subunit | 0.427 | 2.14E-02 |
| b3748 | *rbsD* | ribose pyranase | 0.426 | 1.66E-03 |
| b3575 | *yiaK* | 2,3-diketo-L-gulonate reductase | 0.424 | 2.27E-02 |
| b3214 | *gltF* | periplasmic protein | 0.419 | 1.42E-02 |
| b3666 | *uhpT* | UhpT-hexose phosphate MFS transporter | 0.418 | 1.16E-02 |
| b1901 | *araF* | arabinose ABC transporter - periplasmic binding protein | 0.417 | 2.75E-04 |
| b1615 | *uidC* | membrane-associated protein | 0.417 | 1.28E-02 |
| b3905 | *rhaS* | RhaS transcriptional activator | 0.416 | 1.50E-03 |
| b3240 | *aaeB* | AaeAB Hydroxylated, Aromatic Carboxylic Acid Efflux Transport System Protein B | 0.415 | 1.91E-04 |
| b2841 | *araE* | AraE arabinose MFS transporter | 0.414 | 1.19E-04 |
| b3926 | *glpK* | glycerol kinase | 0.413 | 5.48E-05 |
| b3132 | *kbaZ* | tagatose 6-phosphate aldolase 1, kbaZ subunit | 0.412 | 1.99E-03 |
| b4067 | *actP* | acetate / glycolate transporter | 0.406 | 2.08E-02 |
| b0108 | *ppdD* | prepilin peptidase dependent protein | 0.406 | 3.23E-02 |
| b3428 | *glgP* | glycogen phosphorylase | 0.405 | 8.53E-04 |
| b3072 | *aer* | aerotaxis sensor receptor, flavoprotein | 0.404 | 5.23E-06 |
| b0034 | *caiF* | CaiF transcriptional activator | 0.402 | 3.09E-02 |
| b2707 | *srlR* | GutR DNA-binding transcriptional repressor | 0.400 | 2.14E-02 |
| b2943 | *galP* | GalP - galactose MFS transporter | 0.400 | 2.12E-02 |
| b2799 | *fucO* | L-1,2-propanediol oxidoreductase | 0.399 | 7.56E-06 |
| b0553 | *nmpC* | outer membrane porin protein; locus of qsr prophage | 0.386 | 2.06E-03 |
| b4122 | *fumB* | fumarase B | 0.386 | 1.26E-03 |
| b4213 | *cpdB* | 2',3'-cyclic nucleotide 2'-phosphodiesterase / 3'-nucleotidase | 0.379 | 4.82E-04 |
| b4118 | *melR* | MelR DNA-binding transcriptional dual regulator | 0.379 | 1.66E-06 |
| b2143 | *cdd* | cytidine deaminase | 0.379 | 3.78E-06 |
| b3366 | *nirD* | nitrite reductase, small subunit | 0.378 | 2.80E-03 |
| b3116 | *tdcC* | TdcC threonine STP transporter | 0.376 | 1.71E-03 |
| b3934 | *cytR* | CytR DNA-binding transcriptional repressor | 0.376 | 6.14E-04 |
| b3413 | *gntX* | protein involved in utilization of DNA as a carbon source | 0.363 | 3.41E-03 |
| b3723 | *bglG* | BglG transcriptional antiterminator | 0.361 | 2.77E-03 |
| b3709 | *tnaB* | TnaB tryptophan ArAAP transporter | 0.354 | 1.78E-02 |
| b3925 | *glpX* | fructose 1,6-bisphosphatase II | 0.353 | 6.75E-05 |
| b2240 | *glpT* | GlpT glycerol-3-P MFS transporter | 0.351 | 3.87E-04 |
| b2706 | *gutM* | GutM DNA-binding transcriptional activator | 0.350 | 1.11E-02 |
| b1892 | *flhD* | DNA-binding transcriptional dual regulator with FlhC | 0.345 | 3.13E-05 |
| b4003 | *zraS* | ZraS sensory histidine kinase | 0.333 | 2.05E-03 |
| b3514 | *mdtF* | MdtEF-TolC multidrug efflux transport system - permease subunit | 0.330 | 6.08E-04 |
| b2468 | *aegA* | putative oxidoreductase, Fe-S subunit | 0.327 | 1.72E-05 |
| b1002 | *agp* | 3-phytase / glucose-1-phosphatase | 0.327 | 7.89E-05 |
| b2241 | *glpA* | glycerol-3-phosphate dehydrogenase (anaerobic), large subunit | 0.327 | 1.75E-05 |
| b3114 | *tdcE* | 2-ketobutyrate formate-lyase/pyruvate formate-lyase 4, inactive | 0.325 | 1.77E-02 |
| b3394 | *hofN* | protein involved in utilization of DNA as a carbon source | 0.321 | 1.71E-02 |
| b0113 | *pdhR* | PdhR DNA-binding transcriptional dual regulator | 0.317 | 3.44E-11 |
| b3415 | *gntT* | GntT Gluconate Gnt transporter | 0.316 | 9.34E-05 |
| b4291 | *fecA* | outer membrane receptor; citrate-dependent iron transport, outer membrane receptor | 0.314 | 6.17E-03 |
| b1516 | *lsrB* | AI-2 ABC transporter - periplasmic binding protein | 0.306 | 1.34E-02 |
| b2488 | *hyfH* | hydrogenase 4, component H | 0.303 | 4.14E-03 |
| b4267 | *idnD* | L-idonate 5-dehydrogenase | 0.300 | 7.75E-03 |
| b1734 | *chbF* | diacetylchitobiose-6-phosphate hydrolase | 0.299 | 1.05E-02 |
| b4265 | *idnT* | L-idonate / 5-ketogluconate / gluconate transporter | 0.291 | 5.43E-04 |
| b3117 | *tdcB* | catabolic threonine dehydratase | 0.290 | 5.72E-03 |
| b3093 | *exuT* | ExuT hexuronate MFS transporter | 0.287 | 2.96E-04 |
| b3224 | *nanT* | NanT sialic acid MFS transporter | 0.283 | 1.25E-04 |
| b4196 | *ulaD* | 3-keto-L-gulonate 6-phosphate decarboxylase | 0.281 | 9.08E-05 |
| b2663 | *gabP* | GabP APC transporter | 0.281 | 5.40E-06 |
| b4266 | *idnO* | 5-keto-D-gluconate 5-reductase | 0.280 | 5.20E-05 |
| b3452 | *ugpA* | glycerol-3-phosphate / glycerol-2-phosphate ABC transporter - putative membrane subunit | 0.276 | 1.83E-02 |
| b0333 | *prpC* | methylcitrate synthase | 0.272 | 4.32E-02 |
| b3927 | *glpF* | GlpF glycerol MIP channel | 0.271 | 1.57E-05 |
| b3708 | *tnaA* | L-cysteine desulfhydrase / tryptophanase | 0.268 | 2.54E-02 |
| b3566 | *xylF* | xylose ABC transporter - periplasmic binding protein | 0.266 | 1.81E-02 |
| b3513 | *mdtE* | MdtEF-TolC multidrug efflux transport system - membrane fusion protein | 0.263 | 5.28E-03 |
| b3571 | *malS* | α-amylase | 0.262 | 2.83E-04 |
| b3426 | *glpD* | glycerol 3-phosphate dehydrogenase, aerobic | 0.261 | 5.22E-06 |
| b0107 | *hofB* | protein involved in plasmid replication | 0.260 | 2.80E-03 |
| b4195 | *ulaC* | L-ascorbate-specific enzyme IIA component of PTS | 0.258 | 1.00E-03 |
| b3393 | *hofO* | protein involved in utilization of DNA as a carbon source | 0.257 | 2.62E-02 |
| b2095 | *gatZ* | D-tagatose 1,6-bisphosphate aldolase 2, subunit | 0.256 | 9.16E-07 |
| b3752 | *rbsK* | ribokinase | 0.255 | 8.74E-03 |
| b1819 | *manZ* | mannose PTS permease - ManZ subunit | 0.251 | 7.18E-06 |
| b3134 | *agaW* | PTS system N-acetylgalactosameine-specific IIC component 2 | 0.250 | 1.81E-02 |
| b3599 | *mtlA* | mannitol PTS permease | 0.249 | 8.93E-06 |
| b3750 | *rbsC* | ribose ABC transporter - membrane subunit | 0.249 | 4.41E-04 |
| b4036 | *lamB* | phage lambda receptor protein; maltose high-affinity receptor | 0.246 | 1.69E-03 |
| b4035 | *malK* | maltose ABC transporter - ATP binding subunit | 0.245 | 3.84E-04 |
| b1415 | *aldA* | aldehyde dehydrogenase A, NAD-linked | 0.239 | 1.48E-03 |
| b3578 | *yiaN* | L-dehydroascorbate transporter | 0.217 | 5.11E-05 |
| b4471 | *tdcG* | L-serine deaminase III | 0.215 | 3.08E-03 |
| b1387 | *paaZ* | oxepin-CoA hydrolase/3-oxo-5,6-dehydrosuberyl-CoA semialdehyde dehydrogenase | 0.212 | 7.08E-05 |
| b3576 | *yiaL* | conserved protein | 0.202 | 3.15E-03 |
| b1101 | *ptsG* | fused glucose-specific PTS enzymes: IIB component/IIC component | 0.202 | 4.96E-08 |
| b3588 | *aldB* | acetaldehyde dehydrogenase | 0.201 | 6.40E-06 |
| b2092 | *gatC* | galactitol-specific enzyme IIC component of PTS | 0.197 | 4.93E-06 |
| b2800 | *fucA* | L-fuculose-phosphate aldolase | 0.187 | 4.69E-04 |
| b1038 | *csgF* | curli assembly component | 0.187 | 4.14E-04 |
| b4032 | *malG* | maltose ABC transporter - membrane subunit | 0.186 | 5.47E-04 |
| b1818 | *manY* | mannose PTS permease - ManY subunit | 0.185 | 2.19E-10 |
| b2096 | *gatY* | D-tagatose 1,6-bisphosphate aldolase 2, catalytic subunit | 0.169 | 5.07E-09 |
| b4310 | *nanM* | N-acetylneuraminate mutarotase | 0.169 | 2.35E-04 |
| b3225 | *nanA* | N-acetylneuraminate lyase | 0.166 | 7.68E-04 |
| b2093 | *gatB* | galactitol-specific enzyme IIB component of PTS | 0.157 | 8.18E-06 |
| b3395 | *hofM* | protein involved in utilization of DNA as a carbon source | 0.157 | 1.05E-04 |
| b1517 | *lsrF* | predicted class I aldolase | 0.154 | 1.15E-02 |
| b1817 | *manX* | mannose PTS permease - ManX subunit | 0.151 | 1.36E-10 |
| b3722 | *bglF* | β-glucoside PTS permease | 0.150 | 5.65E-04 |
| b2705 | *srlD* | sorbitol-6-phosphate dehydrogenase | 0.136 | 6.52E-06 |
| b2703 | *srlE* | glucitol/sorbitol-specific enzyme IIB component of PTS | 0.132 | 5.34E-06 |
| b3670 | *ilvN* | acetolactate synthase I, small subunit | 0.127 | 2.34E-03 |
| b2094 | *gatA* | galactitol-specific enzyme IIA component of PTS | 0.119 | 4.79E-09 |
| b2091 | *gatD* | galactitol-1-phosphate dehydrogenase | 0.106 | 9.33E-09 |
| b2704 | *srlB* | glucitol/sorbitol-specific enzyme IIA component of PTS | 0.088 | 5.25E-05 |
| b2702 | *srlA* | glucitol/sorbitol-specific enzyme IIC component of PTS | 0.072 | 1.08E-08 |

^a^From the EcoCyc database (http://ecocyc.org)

^b^Fold-change in gene expression between E2 and WT (average of duplicate experiments)
