# Supplementary material for: Improving Ethanol Tolerance of Escherichia coli by Rewiring Its Global Regulator cAMP Receptor Protein (CRP)
Source: PLoS One. 2013 Feb 28;8(2):e57628. doi: 10.1371/journal.pone.0057628 (PMC3585226; doi:10.1371/journal.pone.0057628)
Supplement: Table S6 — Common genes that were either up-regulated OR down-regulated in both iE2 and E2 as compared to their controls in the absence of ethanol stress. (DOCX) [file pone.0057628.s007.docx]

**TABLE S6.** Common genes that were either up-regulated OR down-regulated in both iE2 and E2 as compared to their controls in the absence of ethanol stress.

| **b-number** | **Gene** | **Function^a^** | **Fold-change (E2/control)** | **Fold-change (iE2/BW25113)** |
| --- | --- | --- | --- | --- |
| b1493 | *gadB* | glutamate decarboxylase B | 7.246 | 50.525 |
| b1492 | *gadC* | GadC GABA APC transporter | 6.587 | 44.773 |
| b3517 | *gadA* | glutamate decarboxylase A | 2.690 | 20.320 |
| b3512 | *gadE* | GadE DNA-binding transcriptional activator | 3.616 | 18.309 |
| b3513 | *mdtE* | MdtEF-TolC multidrug efflux transport system - membrane fusion protein | 2.827 | 17.156 |
| b3516 | *gadX* | GadX DNA-binding transcriptional dual regulator | 2.078 | 7.209 |
| b2508 | *guaB* | IMP dehydrogenase | 4.841 | 5.066 |
| b0343 | *lacY* | LacY lactose MFS transporter | 2.441 | 4.412 |
| b2507 | *guaA* | GMP synthetase | 4.564 | 3.551 |
| b1038 | *csgF* | curli assembly component | 2.473 | 3.163 |
| b1272 | *sohB* | predicted inner membrane peptidase | 2.822 | 2.513 |
| b2235 | *nrdB* | ribonucleoside diphosphate reductase 1, β subunit dimer | 2.578 | 2.474 |
| b0907 | *serC* | phosphohydroxythreonine aminotransferase / 3-phosphoserine aminotransferase | 2.129 | 2.231 |
| b0430 | *cyoC* | cytochrome bo terminal oxidase subunit III | 2.764 | 2.118 |
| b4067 | *actP* | acetate / glycolate transporter | 0.177 | 0.498 |
| b2492 | *focB* | FocB formate FNT transporter | 0.499 | 0.490 |
| b4197 | *ulaE* | L-xylulose 5-phosphate 3-epimerase | 0.442 | 0.484 |
| b2805 | *fucR* | FucR transcriptional activator | 0.380 | 0.482 |
| b1620 | *malI* | MalI DNA-binding transcriptional repressor | 0.428 | 0.467 |
| b3414 | *nfuA* | iron-sulfur cluster scaffold protein | 0.467 | 0.450 |
| b3905 | *rhaS* | RhaS transcriptional activator | 0.475 | 0.437 |
| b4194 | *ulaB* | L-ascorbate-specific enzyme IIB component of PTS | 0.456 | 0.436 |
| b0113 | *pdhR* | PdhR DNA-binding transcriptional dual regulator | 0.217 | 0.414 |
| b4196 | *ulaD* | 3-keto-L-gulonate 6-phosphate decarboxylase | 0.269 | 0.404 |
| b4213 | *cpdB* | 2',3'-cyclic nucleotide 2'-phosphodiesterase / 3'-nucleotidase | 0.435 | 0.398 |
| b3236 | *mdh* | malate dehydrogenase | 0.396 | 0.390 |
| b2799 | *fucO* | L-1,2-propanediol oxidoreductase | 0.244 | 0.384 |
| b1594 | *dgsA* | DgsA DNA-binding transcriptional repressor | 0.454 | 0.370 |
| b1205 | *ychH* | stress-induced protein | 0.246 | 0.360 |
| b2147 | *preA* | NADH-dependent dihydropyrimidine dehydrogenase subunit | 0.099 | 0.352 |
| b3868 | *glnG* | NtrC transcriptional dual regulator | 0.422 | 0.350 |
| b1415 | *aldA* | aldehyde dehydrogenase A, NAD-linked | 0.159 | 0.339 |
| b4003 | *zraS* | ZraS sensory histidine kinase | 0.418 | 0.335 |
| b3566 | *xylF* | xylose ABC transporter - periplasmic binding protein | 0.217 | 0.330 |
| b1002 | *agp* | 3-phytase / glucose-1-phosphatase | 0.327 | 0.323 |
| b2801 | *fucP* | FucP fucose MFS transporter | 0.433 | 0.313 |
| b2146 | *preT* | NADH-dependent dihydropyrimidine dehydrogenase subunit | 0.123 | 0.310 |
| b4268 | *idnK* | D-gluconate kinase, thermosensitive | 0.221 | 0.298 |
| b4460 | *araH* | arabinose ABC transporter - membrane subunit | 0.211 | 0.285 |
| b3091 | *uxaA* | D-altronate dehydratase | 0.379 | 0.281 |
| b3113 | *tdcF* | predicted L-PSP (mRNA) endoribonuclease | 0.283 | 0.275 |
| b2803 | *fucK* | L-fuculokinase | 0.383 | 0.274 |
| b2614 | *grpE* | phage lambda replication; host DNA synthesis; heat shock protein; protein repair | 0.201 | 0.271 |
| b3221 | *yhcH* | conserved protein | 0.220 | 0.268 |
| b4266 | *idnO* | 5-keto-D-gluconate 5-reductase | 0.267 | 0.267 |
| b3092 | *uxaC* | D-glucuronate isomerase / D-galacturonate isomerase | 0.347 | 0.262 |
| b3565 | *xylA* | xylose isomerase | 0.249 | 0.253 |
| b0679 | *nagE* | N-acetylglucosamine PTS permease | 0.489 | 0.243 |
| b4323 | *uxuB* | D-mannonate oxidoreductase | 0.082 | 0.239 |
| b4069 | *acs* | acetyl-CoA synthetase (AMP-forming) | 0.014 | 0.236 |
| b2365 | *dsdX* | DsdX Gnt tranporter | 0.348 | 0.234 |
| b1817 | *manX* | mannose PTS permease - ManX subunit | 0.369 | 0.220 |
| b3134 | *agaW* | PTS system N-acetylgalactosameine-specific IIC component 2 | 0.360 | 0.218 |
| b3133 | *agaV* | PTS system, cytoplasmic, N-acetylgalactosamine-specific IIB component 2 (EIIB-AGA) | 0.258 | 0.215 |
| b3575 | *yiaK* | 2,3-diketo-L-gulonate reductase | 0.197 | 0.202 |
| b2841 | *araE* | AraE arabinose MFS transporter | 0.245 | 0.198 |
| b4322 | *uxuA* | D-mannonate dehydratase | 0.186 | 0.196 |
| b2802 | *fucI* | L-fucose isomerase | 0.270 | 0.180 |
| b3114 | *tdcE* | 2-ketobutyrate formate-lyase/pyruvate formate-lyase 4, inactive | 0.249 | 0.167 |
| b4311 | *nanC* | N-acetylneuraminic acid outer membrane channel | 0.153 | 0.166 |
| b2092 | *gatC* | galactitol-specific enzyme IIC component of PTS | 0.424 | 0.164 |
| b3528 | *dctA* | DctA dicarboxylate DAACS transporter | 0.179 | 0.162 |
| b0598 | *cstA* | peptide transporter induced by carbon starvation | 0.063 | 0.161 |
| b2091 | *gatD* | galactitol-1-phosphate dehydrogenase | 0.302 | 0.147 |
| b4118 | *melR* | MelR DNA-binding transcriptional dual regulator | 0.308 | 0.144 |
| b2093 | *gatB* | galactitol-specific enzyme IIB component of PTS | 0.457 | 0.136 |
| b2151 | *galS* | GalS DNA-binding transcriptional dual regulator | 0.124 | 0.127 |
| b3927 | *glpF* | GlpF glycerol MIP channel | 0.119 | 0.123 |
| b3576 | *yiaL* | conserved protein | 0.339 | 0.115 |
| b3670 | *ilvN* | acetolactate synthase I, small subunit | 0.301 | 0.114 |
| b4321 | *gntP* | GntP Gluconate Gnt transporter | 0.267 | 0.105 |
| b3077 | *ebgC* | evolved β-D-galactosidase, β subunit | 0.390 | 0.103 |
| b3222 | *nanK* | N-acetylmannosamine kinase | 0.109 | 0.102 |
| b2957 | *ansB* | asparaginase II | 0.473 | 0.101 |
| b0759 | *galE* | UDP-glucose 4-epimerase | 0.001 | 0.099 |
| b3581 | *sgbH* | 3-keto-L-gulonate 6-phosphate decarboxylase | 0.186 | 0.099 |
| b3926 | *glpK* | glycerol kinase | 0.227 | 0.098 |
| b4267 | *idnD* | L-idonate 5-dehydrogenase | 0.167 | 0.093 |
| b3671 | *ilvB* | acetolactate synthase I, large subunit | 0.212 | 0.079 |
| b3115 | *tdcD* | propionate kinase | 0.026 | 0.077 |
| b4032 | *malG* | maltose ABC transporter - membrane subunit | 0.283 | 0.076 |
| b3116 | *tdcC* | TdcC threonine STP transporter | 0.051 | 0.074 |
| b3076 | *ebgA* | evolved β-D-galactosidase, α subunit | 0.210 | 0.071 |
| b4239 | *treC* | trehalose-6-phosphate hydrolase | 0.395 | 0.069 |
| b0596 | *entA* | 2,3-dihydro-2,3-dihydroxybenzoate dehydrogenase | 0.096 | 0.063 |
| b2705 | *srlD* | sorbitol-6-phosphate dehydrogenase | 0.496 | 0.060 |
| b1900 | *araG* | arabinose ABC transporter - ATP binding subunit | 0.131 | 0.056 |
| b4240 | *treB* | fused trehalose(maltose)-specific PTS enzyme: IIB component/IIC component | 0.248 | 0.054 |
| b4033 | *malF* | maltose ABC transporter - membrane subunit | 0.306 | 0.052 |
| b3224 | *nanT* | NanT sialic acid MFS transporter | 0.078 | 0.050 |
| b1901 | *araF* | arabinose ABC transporter - periplasmic binding protein | 0.136 | 0.049 |
| b4037 | *malM* | maltose regulon periplasmic protein | 0.308 | 0.042 |
| b2704 | *srlB* | glucitol/sorbitol-specific enzyme IIA component of PTS | 0.381 | 0.041 |
| b2148 | *mglC* | galactose ABC transporter - membrane subunit | 0.198 | 0.036 |
| b4034 | *malE* | maltose ABC transporter - periplasmic binding protein | 0.284 | 0.034 |
| b2149 | *mglA* | galactose ABC transporter - ATP binding subunit | 0.090 | 0.032 |
| b3118 | *tdcA* | TdcA DNA-binding transcriptional activator | 0.122 | 0.030 |
| b4036 | *lamB* | phage lambda receptor protein; maltose high-affinity receptor | 0.264 | 0.026 |
| b2150 | *mglB* | galactose ABC transporter - periplasmic binding protein | 0.030 | 0.026 |
| b2239 | *glpQ* | glycerophosphoryl diester phosphodiesterase, periplasmic | 0.227 | 0.024 |
| b4035 | *malK* | maltose ABC transporter - ATP binding subunit | 0.266 | 0.022 |
| b3225 | *nanA* | N-acetylneuraminate lyase | 0.132 | 0.020 |
| b3223 | *nanE* | predicted N-acetylmannosamine-6-phosphate epimerase | 0.035 | 0.014 |
| b2240 | *glpT* | GlpT glycerol-3-P MFS transporter | 0.220 | 0.014 |
| b3709 | *tnaB* | TnaB tryptophan ArAAP transporter | 0.039 | 0.012 |
| b3117 | *tdcB* | catabolic threonine dehydratase | 0.040 | 0.008 |
| b3708 | *tnaA* | L-cysteine desulfhydrase / tryptophanase | 0.019 | 0.004 |

^a^From the EcoCyc database (http://ecocyc.org)
