# Supplementary material for: Improving Ethanol Tolerance of Escherichia coli by Rewiring Its Global Regulator cAMP Receptor Protein (CRP)
Source: PLoS One. 2013 Feb 28;8(2):e57628. doi: 10.1371/journal.pone.0057628 (PMC3585226; doi:10.1371/journal.pone.0057628)
Supplement: Table S7 — Common genes that were either up-regulated OR down-regulated in both iE2 and E2 as compared to their controls in the presence of ethanol stress. (DOCX) [file pone.0057628.s008.docx]

**TABLE S7.** Common genes that were either up-regulated OR down-regulated in both iE2 and E2 as compared to their controls in the presence of ethanol stress.

| **b-number** | **Gene** | **Function^a^** | **Fold-change (E2/control)** | **Fold-change (iE2/BW25113)** |
| --- | --- | --- | --- | --- |
| b0759 | *galE* | UDP-glucose 4-epimerase | 265.076 | 22.301 |
| b0431 | *cyoB* | cytochrome bo terminal oxidase subunit I | 2.641 | 3.661 |
| b1531 | *marA* | MarA DNA-binding transcriptional dual regulator | 2.700 | 3.072 |
| b0429 | *cyoD* | cytochrome bo terminal oxidase subunit IV | 3.050 | 2.561 |
| b1112 | *bhsA* | protein involved in stress resistance and biofilm formation | 4.061 | 2.528 |
| b0583 | *entD* | phosphopantetheinyl transferase | 2.089 | 2.387 |
| b1819 | *manZ* | mannose PTS permease - ManZ subunit | 0.251 | 0.477 |
| b2092 | *gatC* | galactitol-specific enzyme IIC component of PTS | 0.197 | 0.459 |
| b4266 | *idnO* | 5-keto-D-gluconate 5-reductase | 0.280 | 0.447 |
| b4118 | *melR* | MelR DNA-binding transcriptional dual regulator | 0.379 | 0.389 |
| b2663 | *gabP* | GabP APC transporter | 0.281 | 0.373 |
| b3367 | *nirC* | NirC nitrite FNT transporter | 0.476 | 0.373 |
| b4194 | *ulaB* | L-ascorbate-specific enzyme IIB component of PTS | 0.453 | 0.281 |
| b1101 | *ptsG* | fused glucose-specific PTS enzymes: IIB component/IIC component | 0.202 | 0.233 |
| b2703 | *srlE* | glucitol/sorbitol-specific enzyme IIB component of PTS | 0.132 | 0.062 |
| b2702 | *srlA* | glucitol/sorbitol-specific enzyme IIC component of PTS | 0.072 | 0.028 |
| b2705 | *srlD* | sorbitol-6-phosphate dehydrogenase | 0.136 | 0.026 |
| b2704 | *srlB* | glucitol/sorbitol-specific enzyme IIA component of PTS | 0.088 | 0.025 |

^a^From the EcoCyc database (http://ecocyc.org)
